# A complex survivorship intervention utilizing electronic patient-reported outcomes in breast and gynecologic Cancer: the linking you to support and advice [*LYSA]* trial

**DOI:** 10.1016/j.breast.2026.104740

**Published:** 2026-02-19

**Authors:** Katie E. Johnston, Kate O'Connell, Laia Raigal-Aran, Samantha J. Cushen, Andrea Davis, Fiona Byrne, Naoimh Flynn, Seamus O'Reilly, Sinead Noonan, Dearbhaile C. Collins, Vicki M. Cleary, Maccon Keane, Noreen Kearns, Megan McCarthy, Katarina Medved, Mohammad Javad Ghassemi Rad, Aileen Murphy, Veronica McInerney, Aoife Lowery, Brendan Palmer, Darren Dahly, Roisin M. Connolly, Josephine Hegarty

**Affiliations:** a Cancer Research @UCC, College of Medicine and Health, University College Cork, Cork, Ireland; b CUH/UCC Cancer Centre, Cork University Hospital, Cork, Ireland; c Clinical Nutrition and Oncology Research Group, School of Food and Nutritional Sciences, University College Cork, Cork, Ireland; d Department of Clinical Nutrition and Dietetics, Cork University Hospital, Cork, Ireland; e Department of Medical Oncology, South Infirmary Victoria University Hospital, Cork, Ireland; f Department of Gynecology Oncology, Cork University Maternity Hospital, Cork, Ireland; g Department of Gynecology Oncology, University Hospital Kerry, Kerry, Ireland; h Department of Oncology, Galway University Hospital, Galway, Ireland; i Catherine McAuley School of Nursing and Midwifery, University College Cork, Cork, Ireland; j Department of Economics, Cork University Business School, University College Cork, Cork, Ireland; k HRB Clinical Research Facility, University of Galway, Galway, Ireland; l HRB Clinical Research Facility, School of Public Health, University College Cork, Cork, Ireland; m School of Public Health, University College Cork, Cork, Ireland

**Keywords:** Breast neoplasms, Gynecologic neoplasms, Patient reported outcome measures, Feasibility studies, Survivorship, Allied health care professionals

## Abstract

**Purpose:**

Those living beyond a cancer diagnosis experience unmet supportive care needs due to fragmented post-treatment pathways and limited integration of digital health tools. The Linking You to Support and Advice (LYSA) trial assessed the feasibility of a complex-intervention which incorporated a nurse- and dietitian-led multidisciplinary clinic and a digital platform for capturing and responding to electronic Patient-Reported Outcome (ePRO) data for women with early-stage breast and gynecologic cancer less than 12 months post-primary curative therapy.

**Methods:**

The LYSA trial was an unblinded, randomized, controlled, feasibility trial co-designed with public and patient involvement, conducted across two cancer centers in Ireland. Participants were randomized to the experimental arm, receiving bi-monthly ePRO assessments and trigger-initiated responses to ePROs for 12 months; or the active comparator arm, receiving usual care. Primary feasibility outcomes included participant enrolment, ePRO survey completion, and healthcare professional engagement triggered by ePRO assessments. Secondary outcomes focused on symptom scores, health-related quality of life (HRQOL), and patient satisfaction. A process evaluation explored factors affecting implementation.

**Results:**

The trial met its three predefined feasibility outcomes: 200 participants were enrolled (84% breast, 16% gynecologic), >85% of baseline and endpoint surveys were completed, and >70% of participants in the experimental arm engaged in nurse and dietetic consultations following ePRO symptom triggers. The experimental arm demonstrated significant improvements in fatigue (*p = 0.018*), anxiety (*p = 0.012*), depression (*p < 0.001*) and HRQOL (*p = 0.031*) scores. The process evaluation indicated high levels of satisfaction with the intervention, with positive feedback on the multidisciplinary approach and responsive symptom management.

**Conclusions:**

LYSA demonstrates the feasibility and acceptability of an ePRO-led survivorship approach, with potential HRQOL and symptom benefits, warranting a powered efficacy trial.

## Highlights

• LYSA, a trial of a complex survivorship intervention incorporating ePROs, met its primary feasibility endpoint.
• 200 participants enrolled and 86.5% completed baseline and end of study surveys.
• 84% (experimental) and 72% (active comparator) participants undertook nurse/dietitian consultations following ePRO triggers.
• Process evaluation found the intervention was highly acceptable to participants and healthcare professionals.
• The experimental arm experienced significantly improved quality of life compared to the active comparator arm.

## 1 Introduction

Breast, cervical and endometrial cancers are within the top six female cancer cases diagnosed globally [1]. In Ireland, female cancers (breast, cervical and endometrial) have relatively high overall 5-year net survival rates, currently at 88%, 65% and 78% respectively [2]. The estimated number of new cases of female cancers from 2022 to 2045 is projected to increase from 9.66 million to 15.2 million globally [3]. Hormone-receptor positive breast cancers account for 70% of all female breast cancers diagnosed [4]. Given the high prevalence of cancer diagnoses and unmet supportive care needs [5], there is a pressing need for coordinated care to ensure optimal patient outcomes and quality of life beyond the completion of primary treatment.

Despite recommendations from international oncology guidelines [[6], [7], [8], [9], [10], [11]], standardized and individualized approaches to post-treatment supportive care are lacking; with a recent umbrella review (n = 123,411 participants) demonstrating that individuals affected by cancer continue to report that healthcare systems are not meeting their supportive care needs [12]. Effective supportive care pathways should aim to improve outcomes in a sustainable, cost-effective and equitable manner while minimizing the burdens on patients, caregivers and clinicians [13]. Supportive care spans health promotion, chronic condition management, surveillance for recurrent and new malignancies, and the mitigation of physical and psychological effects of cancer and its treatment [14]. Foundational frameworks by Nekhlyudov et al. (2019) [14] and Alfano et al. (2016) [15] have outlined principles for the delivery of high-quality survivorship care – emphasizing coordination between providers, remote monitoring, triage algorithms and stakeholder engagement; as well as advocating for the integration of evidence-based models across clinical, research, and policy domains [14].

Central to these models is the integration of digital health tools, including telehealth, electronic care plans, and electronic patient reported outcomes (ePROs), to enhance accessibility, personalization and continuity of care [14,15]. These insights have been reinforced in recent literature. Nekhlyudov et al. (2024) highlighted ongoing fragmentation in survivorship care and further underscored the role of digital innovation in enabling real-time symptom tracking, improving communication, and supported shared decision-making [[16], [17], [18], [19]]. Basch et al. (2005) introduced the feasibility of patient-reported symptom monitoring in cancer care, demonstrating improvements in communication, safety and clinical efficiency [20]. Subsequently, Basch and colleagues noted that successful implementation of ePROs depend on process optimization approaches that addresses key factors like software, leadership, workflow, deployment of personnel with roles changes to support ePROs, and patient engagement [21]. Recently, a 2024 systematic review (n = 85 studies) emphasized the importance of innovating and integrating ePRO systems into cancer care pathways, supporting remote monitoring and optimizing patient outcomes [22]. When combined with evidence-based symptom management protocols and multidisciplinary collaboration, ePROs offer a promising approach to optimize outcomes for cancer survivors [23].

Taking cognizance of this background, we report results of a randomized controlled trial (Linking You to Support and Advice: LYSA*,* NCT05035173) that assessed the feasibility of introducing a complex-intervention in two regional cancer centers in Ireland. LYSA incorporated a nurse- and dietitian-led multidisciplinary clinic with ePRO technology to regularly collect and action symptom data for women with early-stage hormone receptor-positive breast, endometrial or cervical cancer. Data on usability, satisfaction and overall experience of the LYSA intervention and trial was collected as part of a mixed methods process evaluation.

## 2 Methods and materials

### 2.1 Patient and public involvement

A patient and public involvement (PPI) panel (n = 6), and clinical and academic stakeholders (n = 59) actively contributed to study design, implementation, and dissemination [24]. Any patient-facing instrument was reviewed by the PPI panel, including surveys and symptom pathways. The research team and the PPI panel engaged in quarterly virtual meetings and received monthly email updates and were involved in the development of the accompanying lay summary [Supplementary Fig. S1].

### 2.2 Study design and setting

The study protocol has been published previously [25] [Supplementary File: Study Protocol]. In brief, LYSA was an unblinded, randomized, controlled feasibility trial with parallel experimental and active comparator arms. Recruitment across two Irish cancer centers took place from March 24, 2021 to August 23, 2022. Standardized reporting was supported through the use of the 2010 extension to the CONSORT (consolidated standards of reporting trials) for randomized pilot and feasibility trials [26] [Supplementary Table S1] and the Practical Implementation Sustainability Model-Reach, Effectiveness, Adoption, Implementation, and Maintenance (PRISM -RE-AIM) framework [27].

The study was conducted in accordance with the Declaration of Helsinki and ICH E6 Good Clinical Practices and received full ethical approval from the relevant clinical research ethics committees [CREC Cork Teaching Hospitals ECM 3 December 08, 2020; CREC Galway University Hospitals 121/21], with subsequent amendments approved as necessary.

### 2.3 Eligibility criteria

Eligible participants included women with early-stage hormone-receptor positive breast, endometrial or cervical cancer within 12 months of completing primary curative therapy and with access to the internet. Participants were screened in medical oncology clinics. Consecutive eligible patients were approached. Eligibility was confirmed, and written consent was obtained by the research nurse, in person or remotely.

### 2.4 Intervention

The LYSA trial investigated the effect of a supportive care intervention, provided in response to changes in ePROs on HRQOL and symptoms experienced. Data was collected via the Castor Electronic Data Capture [EDC] platform [28].

**Fig. 1:**
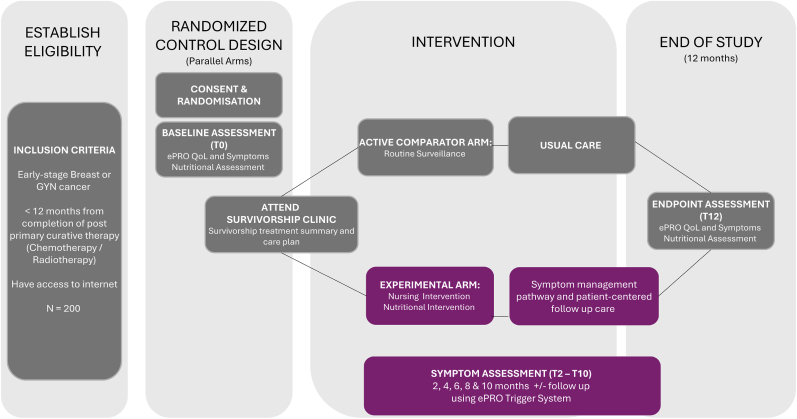
LYSA Trial Schema.

Participants in the experimental and active comparator arms engaged in two in-person visits to the survivorship clinic (at baseline and end of study) and received a survivorship treatment summary and care plan at baseline (Fig. 1). Both arms completed the ePRO Symptom, QoL and Nutritional Assessment Survey packages at baseline [T0] and end of study [T12] and had access to usual care.

Participants in the experimental arm completed ePROs, including symptom assessment, every 2 months between baseline and end of study (Fig. 1). ePRO survey responses activated an online trigger system that initiated prompt clinical follow-up by the nurse and/or dietitian, including virtual assessments by nurses and/or dietitians and shared symptom management using evidence-based care pathways. The intervention was broadly informed by Symptom Management Theory which views symptom care as a dynamic relationship between symptom experience, symptom management strategies, and associated outcomes [29,30]. Detail of full LYSA intervention is presented in the study protocol [25] and in Supplementary Table S1 in accordance with the TIDieR Framework [31].

### 2.5 Study outcomes

The trial had three predefined primary feasibility outcomes: (1) 200 participants enrolled (2); >50% of baseline and end of study ePRO surveys completed (3); following ePRO symptom triggers, >50% of participants engaged in nurse and dietetic consultations.

Secondary outcomes (exploratory efficacy outcomes) addressed in this manuscript relate to changes in electronic patient-reported symptom scores and HRQOL scores. Process evaluation outcomes were patient reported usability and satisfaction scores. Qualitative experiential data was also collected.

### 2.6 Additional measures

The Symptom Survey ePRO package used items from the Patient Reported Outcome Measurement Information System (PROMIS) [32] and Common Terminology Criteria for Adverse Events (PRO-CTCAE) [33] to assess symptoms. In addition, fear of cancer recurrence, self-reported adjuvant endocrine therapy medication adherence, nutritional status and direct healthcare resource use were measured using researcher designed instruments and will be reported in future manuscripts. HRQOL was assessed using The European Organization for Research and Treatment of Cancer Quality of Life (EORTC QOL) patient-reported core and disease specific measures (EORTC QLQ C30-Core and EORTC QLQ CX24, EN24, BR23) [[34], [35], [36], [37]]. Access to the electronic platform to complete the self-reported ePRO questionnaires was through a survey link sent to participants as required. A full description of study instruments and measurements are provided in the study protocol [25].

### 2.7 Sample size

The target sample size of 200 participants was a pragmatic figure, based on the desire to recruit the largest possible sample, given the expected number of eligible patients attending cancer centers during the time frame of the study [25] [Supplementary File: Protocol]. It was anticipated that this target sample size would provide a solid basis for identifying barriers to clinical implementation [38] and provide enough information on outcome distributions that would feed into the design of a subsequent, definitive efficacy trial [39,40].

### 2.8 Randomization, allocation concealment and blinding

Randomization, conducted by the study's principal statistician, was stratified by cancer diagnosis in a 1:1 ratio for breast, cervical, and endometrial cancer using a computer-generated randomization list using randomly sized blocks of size 4, 6, or 8 and operationalized via the study electronic data capture platform (Castor EDC). The allocations were concealed until patients were formally enrolled and consented into the trial. From that point forward, the study nurse and dietitian were not blinded to allocation, due to the nature of the intervention, and limited resources available for outcome assessor blinding. The study statistician was blinded until the initial analysis of the study data was completed.

### 2.9 Data management

Castor EDC allowed for data validation and metadata development throughout the study in accordance with the FAIR Guiding Principles for scientific data management and stewardship [41]. Study metadata including data dictionary, data codebook and analysis scripts are available through the Open Science Framework repository (https://osf.io/y4wth/). Data sharing ethical approvals provide for future secondary data use in cancer survivorship research.

Requests for pseudonymized data access will be considered by the authors and in line with the General Data Protection Regulation (GDPR) and the Irish Health Research Regulations 2018 [42].

### 2.10 Statistical methods

The feasibility endpoints of 1) percentage of baseline and endpoint surveys completed, and 2) percentage of participants engaged in nurse and dietetic consultations following ePRO symptom triggers were reported alongside 95% Wilson confidence intervals (CIs) [43] and compared to pre-registered progression criteria. Between-arm differences in the end-of-study EORTC QLQ-C30 were estimated using complete-case samples using ordinal regression models with a logit link (i.e. a proportional odds model) [44]. Models were adjusted for baseline scores, and estimated effects were reported as odds-ratios (ORs) where values > 1 reflect better scores in the experimental arm relative to the active comparator arm. All effect estimates were reported alongside 95% CIs. Due to the exploratory nature of these analyses, there was no effort to adjust for multiplicity. PROMIS symptom scores were similarly analyzed but coded in a manner such that ORs <1 reflected a benefit in the experimental arm.

All analyses were conducted using the R statistical programming language (version 4.4.1) [45] and the RStudio IDE (version 2024.12.0). Ordinal regression models were fit using the ordinal package [46]. Other key packages included gtsummary [47] likert [48] and ggplot2 [49].

### 2.11 Process evaluation

Embedded process evaluations are increasingly integrated into trials of health service interventions, offering crucial insights into the underlying processes; to clarify how interventions work, helping to interpret and contextualize study findings [50,51]. An in-depth qualitative descriptive approach, using semi-structured interviews and focus groups, was employed to explore experiences of the LYSA intervention and trial processes. Patients, healthcare professionals, and broader stakeholders involved in the intervention were invited to participate in individual or focus group interviews post-trial. Through the process evaluation the team sought to unpack the complexities of implementing LYSA in a real-world context.

Interviews, conducted by an independent researcher (NK) via MS Teams, lasted 15–60 minutes, were audio-recorded with consent, transcribed, anonymized, and securely stored. Open-ended questions facilitated detailed exploration of participants' views on the clinic, challenges, benefits, and future potential [Supplementary Table S2]. An online survey distributed via Castor EDC software to trial participants captured additional quantitative data on usability and satisfaction, enabling triangulation of findings.

Transcripts were analyzed inductively using Graneheim and Lundman's qualitative content analysis [52], which involved systematically reading raw data to create codes (labels for meaning units), grouping similar codes into subcategories, and then further organizing these into broader categories to reveal patterns or themes emerging from the data without preconceived theories. Separately, the PRISM (RE-AIM) framework was used to deductively organize implementation factors [27]. Rigor was supported through peer validation, rich contextual description, use of exemplar quotes, and adherence to principles of credibility, transferability, dependability, and confirmability [53].

## 3 Results

### 3.1 Participant characteristics

**Fig. 2:**
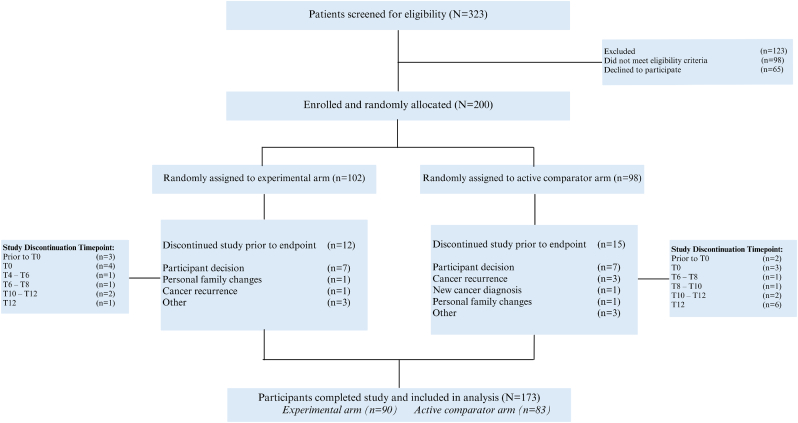
LYSA CONSORT diagram.

Of 323 potential participants screened, 200 women were randomized to the trial across two sites, meeting the first predefined feasibility outcome (Fig. 2). Baseline clinicopathologic and treatment characteristics by study arm are provided in Table 1 and sociodemographic characteristics in Supplementary Table S3. The timeframe of the trial coincided with the COVID19 global pandemic, and a national public health service (Health Service Executive) cyberattack. COVID19 precipitated the use of virtual consent processes and e-consultations.

**Table 1:** Baseline clinicopathologic and treatment characteristics of all randomized participants.

Characteristic	N	Overall	Active Comparator	Experimental
		N = 200	N = 98	N = 102
Age in years (median, range)	192	54 [23 to 78]	55 [31 to 78]	54 [23 to 75]
Cancer diagnosis	195			
*Breast*		164 (84%)	83/96 (86%)	81/99 (82%)
*Cervical*		13 (6.7%)	5/96 (5.2%)	8/99 (8.1%)
*Endometrial*		18 (9.2%)	8/96 (8.3%)	10/99 (10%)
Menopausal status	195			
*Pre*		61 (31%)	28/96 (29%)	33/99 (33%)
*Peri*		20 (10%)	7/96 (7.3%)	13/99 (13%)
*Post*		110 (56%)	57/96 (59%)	53/99 (54%)
*Unknown*		4 (2.1%)	4/96 (4.2%)	0/99 (0%)
ECOG performance status	194			
*0*		64 (33%)	31/96 (32%)	33/98 (34%)
*1*		125 (64%)	62/96 (65%)	63/98 (64%)
*2*		5 (2.6%)	3/96 (3.1%)	2/98 (2.0%)
Prior Surgery	195	186 (95%)	91/96 (95%)	95/99 (96%)
Prior Radiotherapy	195	192 (98%)	94/96 (98%)	98/99 (99%)
Prior Chemotherapy	194	119 (61%)	57/96 (59%)	62/98 (63%)
Prescribed Endocrine Therapy	195	163 (84%)	83/96 (86%)	80/99 (81%)
*Aromatase Inhibito*r^a^		90/163 (55%)	45/83 (52%)	45/80 (60%)
*Tamoxifen*		59/163 (36%)	32/83 (39%)	27/80 (34%)
*Aromatase Inhibito*r^a^*/Tamoxifen with Ovarian Function Suppression*		14/163 (9%)	6/83 (7%)	8/80 (10%)

Abbreviations: ECOG, Eastern Cooperative Oncology Group.

a Aromatase Inhibitors include: Anastrozole, Exemestane and Letrozole.

A total of 86.5% (n = 173) of participants completed the trial [Fig. 2]. Reasons for study discontinuation included participant decision (n = 18), cancer recurrence (n = 4), new cancer diagnosis (n = 1) and other (n = 4) [Supplementary Table S4].

### 3.2 Primary feasibility results

In addition to enrolling all 200 participants as planned, 96.5% of participants completed the baseline ePRO Symptom Survey package (193/200, 95% CI 93 to 98%), while 89% completed the end of study survey (178/200, 95% CI 84 to 93%), thus exceeding the benchmark of >50% of baseline and endpoint ePRO surveys completed [Supplementary Table S5].

The final predefined requirement to confirm feasibility of the LYSA intervention stipulated that >50% of participants engaged in nurse and dietetic consultations following ePRO symptom triggers. Following an ePRO symptom trigger in the experimental arm [Supplementary Fig. S2], 84% (95% CI 76 to 90%) and 72% (62 to 79%) of participants engaged with nurse and dietetic consultations, respectively. Nurse and dietitian consultations were initiated through three possible mechanisms: symptom(s) trigger (n = 565) [Supplementary Table S6]; patient-initiated request for call back (n = 61); or nurse or dietitian's clinical judgement based on overall symptom profile (n = 0).

There were 322 nurse virtual visits initiated by symptom triggers (involving 86 participants), and a further 47 virtual nurse visits due to patient-initiated request for call back (27 participants). In relation to dietetic consultations, there were 243 virtual visits initiated by symptom triggers (73 participants), and a further 14 virtual visits due to patient-initiated request for call back (11 participants).

The median length of in-person nurse visits was 60 minutes (interquartile range [IQR] 45-60), while it was 90 minutes for in-person dietitian visits (IQR 70-120). For symptom-trigger initiated consultations (T2-T10), the median length of a nurse consultation was 35 minutes (IQR 20-60), and for dietitian consultations, 40 minutes (IQR 30-75). The trigger-initiated consultations (T2-T10) equated to 39.1 hours of nursing services and 57.8 hours of dietetic services.

There were 28 medical reviews required by 25 participants, that were prompted by consultation with the research nurse; mean time to medical consultation was 2.4 days (standard deviation (SD) ±8.8; range [days] 0-46). Reasons for medical review included the need for physical examination based on reported symptoms (n = 5); new (n = 2) or worsening (n = 8) symptoms requiring physician input; the need for endocrine therapy (n = 2) or supportive medication prescriptions (n = 8).

### 3.3 Secondary outcomes (exploratory efficacy outcomes)

Between-arm differences in end-of-study EORTC QLQ-C30 scores are shown in Table 2 and disease specific scores in Supplementary Table S7. Participants in the experimental arm experienced better EORTC QLQ-C30 summary scores (*p = 0.031*) and role functioning (*p = 0.026*) when compared to patients in the active comparator arm (baseline-adjusted OR 1.83, 95% CI 1.06 to 3.18; 1.96 (1.09 to 3.58). Fourteen of the 15 remaining sub-scale scores (the outlier being appetite loss) had treatment effect point-estimates that suggested a benefit for the experimental arm relative to active comparator, though 95% CIs were typically wide.

**Table 2:** Standardized EORTC-C30 and PROMIS scores at study end (T12) with estimated treatment effects.

Characteristic		Study Arm (N = 162)		Odds ratio (95% CI)^b^	p-value
		Active Comparator	Experimental		
		N = 76^a^	N = 86^a^		
**Standardized EORTC-C30**					
EORTC C30 global health status score		53 (18); 50 [42, 67]	55 (18); 50 [50, 67]	1.26 (0.72 to 2.2)	0.42
EORTC C30 physical functioning score		81 (20); 87 [73, 93]	86 (14); 87 [80, 100]	1.7 (0.97 to 3.03)	0.067
EORTC C30 role functioning score		68 (32); 67 [50, 100]	79 (23); 83 [67, 100]	1.96 (1.09 to 3.58)	**0.026∗**
EORTC C30 emotional functioning score		67 (23); 71 [50, 83]	74 (17); 75 [67, 92]	1.61 (0.92 to 2.82)	0.098
EORTC C30 cognitive functioning score		67 (28); 67 [50, 83]	70 (22); 67 [50, 83]	1.27 (0.72 to 2.25)	0.42
EORTC C30 social functioning score		73 (25); 67 [67, 100]	79 (20); 83 [67, 100]	1.66 (0.93 to 2.99)	0.088
EORTC C30 fatigue score		64 (22); 67 [56, 78]	69 (20); 67 [56, 89]	1.57 (0.9 to 2.75)	0.11
EORTC C30 nausea and vomiting score		90 (18); 100 [83, 100]	94 (10); 100 [83, 100]	1.58 (0.78 to 3.22)	0.2
EORTC C30 pain score		68 (28); 67 [50, 100]	73 (25); 83 [50, 100]	1.46 (0.83 to 2.56)	0.19
EORTC C30 dyspnea score		83 (23); 100 [67, 100]	86 (18); 100 [67, 100]	1.1 (0.55 to 2.17)	0.79
EORTC C30 insomnia score		59 (32); 67 [33, 100]	62 (26); 67 [33, 67]	1.52 (0.84 to 2.78)	0.17
EORTC C30 appetite loss score		89 (21); 100 [100, 100]	89 (20); 100 [67, 100]	0.77 (0.35 to 1.66)	0.5
EORTC C30 constipation score		77 (27); 100 [67, 100]	82 (26); 100 [67, 100]	1.72 (0.9 to 3.33)	0.1
EORTC C30 diarrhea score		89 (22); 100 [67, 100]	92 (16); 100 [100, 100]	1.47 (0.67 to 3.26)	0.34
EORTC C30 financial problems score		80 (28); 100 [67, 100]	84 (24); 100 [67, 100]	1.93 (0.92 to 4.12)	0.084
EORTC C30 summary score		75 (16); 77 [67, 88]	80 (13); 82 [71, 89]	1.83 (1.06 to 3.18)	**0.031∗**

	**N**	**Active Comparator** **N = 82**^a^	**Experimental** **N = 90**^a^	**Odds ratio** **(95% CI)**^c^	**p-value**

**Standardized PROMIS Scores**					
Fatigue	172	53 (10); 52 [46, 61]	50 (9); 50 [46, 57]	0.53 (0.31 to 0.89)	**0.018 ∗**
Vaginal Discomfort	74	48 (7); 48 [45, 51]	46 (7); 48 [41, 51]	1.17 (0.43 to 3.24)	0.754
Missing Values d		46	52		
Anxiety	172	57 (9); 58 [51, 63]	53 (8); 56 [48, 58]	0.5 (0.29 to 0.86)	**0.012 ∗**
Depression	172	53 (9); 54 [40, 59]	48 (8); 45 [40, 56]	0.36 (0.2 to 0.64)	**< 0.001 ∗**

Abbreviations: CI, Confidence Intervals; EORTC, European Organization for Research and Treatment of Cancer; PROMIS, Patient-Reported Outcomes Measurement Information System.

∗p ≤ 0.05.

a Mean (SD); Median [Q1, Q3].

b Treatment effect estimated using complete-case, baseline-adjusted ordinal regression.

c Treatment effect estimated using complete-case, baseline-adjusted ordinal regression; OR < 1 suggests benefit in the experimental arm relative to the active comparator arm.

d Missing values (n = 98) denote participants who had not had sexual intercourse in the last 30 days.

Between-arm differences in end-of-study PROMIS symptom scores are shown in Table 2. Treatment effect estimates suggested lower, better symptom scores for fatigue (OR 0.53, 95% CI 0.31 to 0.89), anxiety (0.5, 0.29 to 0.86) and depression (0.36, 0.2 to 0.64), but not for vaginal discomfort, in the experimental arm relative to the active comparator arm.

### 3.4 Process evaluation

Qualitative interviews were conducted with twenty study participants, eleven study team members/stakeholders and seven PPI panel members. Study participants were women with breast cancer from the experimental arm (n = 14) and active comparator arm (n = 6). Notably, whilst open to all participants, no patients with gynecological cancers participated in the process evaluation interviews. Two categories and 12 sub-categories are presented in Table 3, which highlight the positive and negative aspects of the LYSA intervention and trial and provide recommendations for future implementation.

**Table 3:** LYSA process evaluation categories, sub-categories and codes.

Main categories a	Sub-categories	Codes
**Positive and negative aspects of the LYSA intervention**	**Enhanced supportive care services different from usual care**	Intervention addresses the sudden reduction in contact/support at the end of treatment, at a time when the patient is vulnerable Intervention provides pathway for managing symptoms and issues Clinic services in one place
	**Positive aspects of the LYSA clinic**	Mindset of survivorship rather than hospital and treatment, helping individuals to live better beyond cancer Supported connecting out (from hospital); accessible from own home and feeling connected in (to hospital) Felt supported, supportive and practical intervention Tailored to individual needs Ensures continuity of care Positive impact on health and wellbeing Timing of intervention right Well-run clinic
	**Positive aspects of the LYSA serial electronic surveys**	Helped to link in, someone available to review Breadth of survey- coverage of symptoms broad Self-check-in with surveys Progress recorded, giving confidence Easy to do and user friendly Quick, no pressure
	**Positive aspects of LYSA *t*rigger system**	The trigger system works well; the dietitian and nurse can follow a patient together Patient can organize joint visits with dietician and nurses Facilitates MDT referral pathway.
	**Positive aspects of managing symptoms**	Early management of symptoms and side effects of treatments outside of usual surveillance focused pathway e.g. tamoxifen or dealing with symptoms that worsened over time Provided a mechanism of sharing details on topics and issues that were perceived to be difficult to talk about e.g. sharing difficulties with sexual health Provided advice and support for ongoing symptoms and issues e.g. lymphoedema Provided a solution when some [patients] perceived that their GP [family physician] was not familiar with or understanding of symptoms Affirmation of having symptoms and issues listened to
	**Other positive aspects**	Promoted focus on health and well being Kept patients out of the emergency department and hospital
	**Negative aspects of the LYSA survey**	Some unclear survey questions- perception [by some] that there was a right answer Repetition and relevance of some survey items Dietetic baseline and end of study assessments take a lot of time to complete
	**Negative aspects of LYSA trigger system**	Time taken to review triggers and identify those triggers that must be seen by a health care professional Sensitivity and specificity of some triggers: some triggers impacted by multi-morbidities (e.g. irritable bowel disease); lack of triggers for person below healthy BMI or in the healthy BMI range but gaining weight
	**Negative aspects of LYSA clinic**	No specific LYSA team support at weekends or at night Symptom management pathways too generic Negative aspects of onward referrals e.g. increased waiting time for patients accessing certain services
	**Positive aspects of LYSA HCPs**	Built relationships with nurse and dietician over time Nurse – “the go to” person; available, knowledgeable, kind person who can give extra advice/support, assist with system navigation to sort issues out Patients felt that they had someone to confide in; a champion
		Dietitians were educators, providing good, practical nutritional advice and resources e.g. cookbook to help patients make changes in their diet Lots of support and information
	**Negative aspects of LYSA HCPs**	More empathy for where the patient is at in their individual journey
**Recommendations**	**Clinic and Survey and services**	Timing of clinic- start 2-3 months after treatments completed, and continue for longer, maintain contact for longer than one year with access based upon need Clinic- include more face-to-face options, perhaps consider a community-based setting Review survey to reduce repetition and length Consider paper and digital surveys for those with limited digital capabilities Refine survey triggering levels and healthcare professional response mechanisms Revise communication/triggering system from using emails to a summary statement/record that can be easily checked within the patients' health record Provide feedback to GP [family physician] Automated feedback to patients on symptoms experienced in survey e.g. visuals or graph to show progress to the patient, provide online feedback Access to more of services/interventions in the community closer to the patient; referral based upon local zip code/GPS or local cancer support services e.g. community physiotherapy services, exercise classes Fully integrated service with everything in one building- ‘One stop shop’ with all services available including genetics for in person visits Be more family centered- include family and children Extend LYSA to include opportunity for a clinic for women with advanced or metastatic cancer More supports re return to work and entitlements More help in navigating mental health e.g. fear of cancer recurrence Increase access to counselling/psychologist/CBT Recommend peer support group to talk/meet others to feel less isolated

Abbreviations: BMI Body Mass Index, CBT Cognitive Behavioral Therapy, GP General Practitioner, GPS Global Positioning System, MDT Multidisciplinary Team.

a Codes, subcategories, and categories emerging from indictive analysis of interview data.

Important findings from the process evaluation include that the LYSA intervention addressed the sudden reduction in contact and support at the end of treatment, at a time when the patient is vulnerable, by providing a clear pathway for managing symptoms and issues. Having a dedicated clinical and ePRO system changes the lens from hospital and treatment to living well beyond cancer. Key positives included patients feeling that they were connected into the hospital from their home; feeling supported giving participants' confidence; feelings of affirmation by having symptoms and issues listened to; and providing continuity of care. One health care professional noted “*They [patient] won't necessarily bring that [decreased libido] up and certainly won't discuss it at a clinical visit …. But in this [survey] it's triggered for an awful lot of women, and it is because they're allowed to ask the question.”*.

Positive and negative implementation factors linked to implementation of the LYSA trial were coded to the PRISM (RE-AIM) framework allowing the team to explore and understand the factors that influenced the implementation of the intervention in a real-world context [Supplementary Table S9]. Key influencing factors included: enabling national policy; service level support; partners and relationships; co-design; funding, resources and infrastructure; digital infrastructure; digital capabilities of patients; and having symptom specific pathways. There was a high degree of consensus among experimental arm participants regarding the intervention's usability and acceptability [Supplementary Table S8]. However, 56% of participants indicated a preference for technology-based questionnaires over traditional paper formats, highlighting both a growing comfort with digital health tools and a cohort of individuals who are more comfortable with paper-based surveys.

## 4 Discussion

This randomized controlled trial of the 12-month LYSA intervention met its three predefined feasibility outcomes, with 200 women enrolled, an 86.5% study completion rate and high engagement with healthcare professionals following ePRO triggers. Predefined secondary objectives also suggested potential efficacy of the LYSA intervention. Notably, favorable outcomes were observed in the experimental arm when compared to the comparator arm, particularly in relation to fatigue, anxiety, and depression symptom scores whilst acknowledging that the study was not powered to test efficacy of the intervention. Furthermore, the LYSA intervention was reported to be highly acceptable to participants, healthcare professionals, PPI partners and stakeholders alike, suggesting that next step investigations of the LYSA intervention can be successfully conducted.

Our findings align with existing research [15,17,22,54] reinforcing established trends and supporting frameworks [14,16,55] for integrating symptom monitoring and ePROs into clinical workflows as a supplement to routine survivorship care. Since the inception of this trial, a key umbrella review of systematic reviews (n = 222 unique studies) evaluating the effects of patient reported outcome measures (PROMs) feedback was published. [56]. Umbrella review authors highlighted PROM feedback consistently enhanced aspects of care processes such as communication and the recognition and management of symptoms, however, the evidence regarding its impact on patient health outcomes was less definitive While 60% (n = 12) of systematic reviews reported promising interventions, results were mixed; particularly for quality of life (n = 7 reported as effective) and psychological symptoms such as anxiety and depression (n = 5 reported unchanged symptoms) [56]. The LYSA intervention suggests preliminary efficacy across multiple domains and quality of life. Through the process evaluation, the research team attribute much of this benefit to its multidisciplinary approach, centralized point of contact, and responsive design enabled by targeted ePRO triggers.

A strength of this feasibility trial design was in the inclusion of a process evaluation. The implementation of complex health interventions such as LYSA into dynamic, multi-level healthcare systems, particularly in hospitals, is inherently complex and multifactorial. The integration of a digital workflow adds another layer of complexity, requiring the collaboration of various stakeholders, including clinical professionals, administrative staff, researchers, and community partners. This complexity is evident when considering the positive and negative implementation factors associated with frameworks like PRISM (RE-AIM), which reflect various domains, such as Reach, Efficacy, Adoption, Implementation, and Maintenance.

The LYSA trial represents a significant advancement in digital survivorship care in Ireland, leading to new real-world data in enhanced supportive care. Conducted across two large Irish cancer centers and including participants from both rural and urban areas, the study provides a robust model for scaling ePRO-driven interventions, and aligns with international digital health strategies such as the EU Beating Cancer Plan [55] and the US 21st Century Cures Act [57]. The randomized design has allowed preliminary comparison between arms, which will guide a fully powered randomized controlled trial.

An additional strength of this trial was the formation of a summative one page, traffic light color coded patient-reported symptom list to guide clinical consultations [Supplementary Fig. 2]. This was a practical tool for multidisciplinary teams to monitor symptoms and provide timely intervention to the growing population of cancer survivors. The longitudinal nature of the LYSA intervention and the serial ePRO collection provided a unique picture of the symptom profile and needs. By enabling nurses and dietitians to collaborate effectively, this digital model recognized that symptoms do not occur in isolation but affect physical, psychological, nutritional, and functional status.

The integration of ePROs with electronic health records remains a key challenge. In Ireland, emerging research, such as the LYSA trial, may strengthen the integration of PROMs into a developing national electronic health record. Current literature supports the role of symptom monitoring in enhancing patient engagement, enabling timely interventions, and reducing adverse events among cancer survivors [17,18,54,56,[58], [59], [60]]. However, harvesting large data sets from ePROs within existing hospital systems remains a barrier. Approximately 70% of ePRO systems in survivorship care remain independent of hospital electronic health records, reducing their full potential for system-wide analytics [54]. Looking ahead, the integration of artificial intelligence (AI) driven algorithms and predictive scoring tools could further optimize ePRO use, enabling smarter, more personalized survivorship care. The establishment of a color-coded trigger alert system in the LYSA trial is a welcome step towards generating assisted prompts for timely, targeted and patient-centered survivorship supportive care.

A few limitations to the LYSA study design must also be acknowledged. The trial focused on three female cancer subtypes (breast, cervical, endometrial) with similar symptom profiles and pathways; limiting generalizability to other cancer types. However, the study intervention could easily be investigated in other cancer populations, including metastatic cohorts, using learnings from this study. Secondly, participants required internet access to meet eligibility criteria based on the ePRO platform we utilized, potentially excluding digitally underserved populations. Finally, although all patient-facing materials were co-designed with the LYSA PPI panel, the potential burden of lengthy symptom questionnaires may be a concern for broader implementation.

Whilst evidence is robust for survivorship care interventions similar to LYSA, sustained evidence of their implementation and translation into routine clinical practice is lacking. The LYSA trial has, however, informed and prioritized survivorship care in these regional cancer centers in Ireland. Future LYSA trial outputs will include an economic analysis, providing data for service development and strategic planning to assist in meeting growing demands for supportive care on the cancer journey. Future research should explore how AI-generated responses, predictive scores, and adaptive algorithms, overseen by clinical experts, can further optimize ePRO systems within cancer centers. By leveraging digitalization, ePROs can inform more inclusive, targeted, efficient and data-driven survivorship care for future generations.

To conclude, the LYSA trial met its primary feasibility endpoint, demonstrating that a complex survivorship intervention incorporating ePROs is feasible and acceptable in routine follow-up care for patients with early-stage breast and gynecologic cancer. High completion rates of ePRO surveys and substantial engagement in symptom-triggered healthcare consultations were observed. The experimental arm showed better symptom management and QOL outcomes, suggesting potential benefits of the intervention. In line with the Medical Research Council's guidance on the development and testing of complex interventions [50], a future definitive randomized controlled trial should build on the outcomes of the LYSA feasibility trial.

## CRediT authorship contribution statement

**Katie E. Johnston:** Writing – review & editing, Writing – original draft, Visualization, Resources, Project administration, Methodology, Investigation, Data curation. **Kate O'Connell:** Writing – review & editing, Writing – original draft, Visualization, Resources, Project administration, Methodology, Investigation, Data curation. **Laia Raigal-Aran:** Writing – review & editing, Writing – original draft, Software, Resources, Project administration, Methodology, Investigation, Data curation. **Samantha J. Cushen:** Writing – review & editing, Supervision, Resources, Methodology. **Andrea Davis:** Writing – review & editing, Resources, Methodology, Investigation. **Fiona Byrne:** Writing – review & editing, Resources, Methodology. **Naoimh Flynn:** Writing – review & editing, Resources, Project administration, Methodology, Investigation, Data curation. **Seamus O'Reilly:** Writing – review & editing, Resources, Methodology. **Sinead Noonan:** Writing – review & editing, Resources, Methodology. **Dearbhaile C. Collins:** Writing – review & editing, Resources, Methodology. **Vicki M. Cleary:** Writing – review & editing, Resources, Methodology. **Maccon Keane:** Writing – review & editing, Resources. **Noreen Kearns:** Writing – review & editing, Resources, Methodology, Data curation. **Megan McCarthy:** Writing – review & editing, Resources, Data curation. **Katarina Medved:** Writing – review & editing, Resources, Investigation, Data curation. **Mohammad Javad Ghassemi Rad:** Writing – review & editing, Resources, Data curation. **Aileen Murphy:** Writing – review & editing, Resources, Methodology. **Veronica McInerney:** Writing – review & editing, Supervision, Resources, Methodology. **Aoife Lowery:** Writing – review & editing, Supervision, Methodology, Funding acquisition. **Brendan Palmer:** Writing – review & editing, Validation, Software, Resources, Project administration, Methodology. **Darren Dahly:** Writing – review & editing, Writing – original draft, Validation, Software, Resources, Project administration, Methodology, Formal analysis. **Roisin M. Connolly:** Writing – review & editing, Writing – original draft, Validation, Supervision, Resources, Methodology, Funding acquisition, Formal analysis, Conceptualization. **Josephine Hegarty:** Writing – review & editing, Writing – original draft, Validation, Supervision, Resources, Methodology, Funding acquisition, Formal analysis, Conceptualization.

## Data availability statement

Due to privacy and ethical restrictions, certain portions of the data may not be publicly available. Interested researchers may contact the corresponding author to discuss access to the data, which will be provided in accordance with applicable data protection regulations and with the approval of the relevant ethics committees.

## Clinical trial Registration

ClinicalTrials.gov, NCT05035173. Retrospectively registered on September 5, 2021.

## Funding Statement

This work is supported by the Irish Cancer Society Women's Health Initiative through the research grants WHI19CON, WHI21COHE (with Pfizer Ireland), WHI22LOW & WHI22COHE. This publication is independent research also supported by Breakthrough Cancer Research (BCR-2019-09-ICS-UCC) and the Health Research Board Ireland (CTIC-2021-002, CRF-2021-005, CRFC-2021-001). Any opinions, findings, conclusions, or recommendations expressed are those of the authors and not necessarily those of the Irish Cancer Society, Breakthrough Cancer Research, Pfizer Ireland or the Health Research Board Ireland.

These funding bodies and the study sponsor (University College Cork) had no role in collecting, analyzing, or interpreting trial data. Any opinions, findings, conclusions, or recommendations expressed in this material are those of the authors and not necessarily those of the funders.

## Conflict of interest

Roisin M Connolly has received research funding for clinical trials from MSD Ireland, Pfizer, Daichii Sankyo, and Astra Zeneca; all to her institution. She has also consulted for Astra Zeneca/Daichii, Gilead, Seagen, and Lilly. She has received travel/conference support from Novartis, Roche and Gilead.

Josephine Hegarty received a research grant from Pfizer to her institution.

Dearbhaile Collins has received honoraria from MSD, Pfizer, Genmab, Seagen, AZD and GSK. She has consulted for Genmab, Seagen and MSD.

Kate O'Connell has received travel/conference support from Novartis, Pfizer and Amgen.

Seamus O'Reilly has received travel support from Novartis, Merck, Daichi Sankyo; and consultancy fees from Roche, Astra Zeneca and Daichi Sankyo.

Sinead Noonan has received research funding for clinical trials from MSD Ireland to her institution, she has received travel/conference support from Roche.

The other authors have no conflicts of interest to disclose.

## Appendix A Supplementary data

Supplementary File: Study Protocol Multimedia component 1
