# Supplementary material for: A complex survivorship intervention utilizing electronic patient-reported outcomes in breast and gynecologic Cancer: the linking you to support and advice [LYSA] trial
Source: Breast. 2026 Feb 19;86:104740. doi: 10.1016/j.breast.2026.104740 (PMC12966741; doi:10.1016/j.breast.2026.104740)
Supplement: Supplementary File: Study Protocol [file mmc1.pdf]

STUDY PROTOCOL

The LYSA Trial

Trial Title: Women's Cancer Survivorship: The LYSA (Linking You to Support and Advice) Trial

Principal Investigator
Professor Roisin Connolly, MB BCh MD
Chair in Cancer Research, University College Cork
Director, Cancer Research @UCC
Western Gateway Building, 4.110
Western Road, Cork, Ireland
T. 021 4922687; M: 087 3427341
E. roisin.connolly@ucc.ie

Co-Principal Investigator
Professor Josephine Hegarty
Head of School of Nursing and Midwifery,
University College Cork
Cork, Ireland
T. 021 4901553; M 087-4177062:
E. J.Hegarty.ucc.ie

Sponsor: University College Cork (UCC) **Sponsor Study Code:** 19137

**Principal Project Reference**	WHI19CON	**Other project references**	**BCR code:** BCR-2019-09-ICS-UCC<br>**ClinicalTrials.gov Identifier:** NCT05035173
**CREC Reference number:**	CUH site:<br>ECM 4 (y) 20/10/2020		
	GUH<br>Ref: 121/21		

Note: previous protocol title "Women's Cancer Survivorship: Supporting and Innovating for Change" (changed on V7)

Protocol Agreement

**Title**	Women's Cancer Survivorship: Supporting and Innovating for Change
**Project Reference**	WHI19CON
**Study Sponsor**	University College Cork (UCC)
**Recruitment Date**	March 2021-March 2022
**Sites**	Cork University Hospital, Cork

I, the undersigned, have read and understand the specific Study Protocol, and agree with the contents. The Study Protocol, the Investigator's Agreements and any additional information provided by the sponsor will serve as a basis for cooperation in the study.

I agree to conduct in person or to supervise the study.

I agree to ensure that all that assist me in the conduct of the study have access to the study Protocol plus any amendments and are aware of their obligations.

Site Principal Investigator:

Professor<br>Roisin Connolly	Roisin Connolly	16-January-2023
Name	Signature	Date

University College Cork/Cork University Hospital
Institution

Name and Address and Professional Position of Principal Investigator:

Professor Roisin Connolly, MB BCh MD
Chair in Cancer Research, University College Cork
Director, Cancer Research @UCC
Western Gateway Building, 4.110
Western Road, Cork, Ireland
T. 021 4922687; M: 087 3427341
E. roisin.connolly@ucc.ie

Summary of study documents versions

Note: *Main Protocol document has its own version and date and each appendix document has its own version and date.*

CREC Reference Number: ECM 4 (y) 20/10/2020

CREC amend Ref	Document	Version	Date	Summary of Changes
N/A	Protocol	V1.0	09 Sep 2020	N/A
N/A	Protocol	V2.0	03 Nov 2020	P18 Specify that participants can chose one to one interview or focus group.<br>Corrected spelling and spacing errors on various pages.
**ECM 3 (ddd) 08/12/2020**	Protocol	V3.0	26 Nov 2020	Clarification of wording: Replaced 'Participants 'with 'Patients' in several places.<br>Indicated Patients and Health Care Professionals (HCPs) where it added clarity.<br>Changed 'Key stakeholders' to 'broader team'<br>Clarified that both patients and HCPs will be invited to a focus group interview or a one-to-one interview, and that they may choose one or the other, or chose not to participate in any interview.
**ECM 3 (r) 09/02/2021**	Protocol	V4.0	8 Jan 2021	**Contacts – Sub-investigators**<br>• Addition of new Research Assistant's name<br>• Modification of list of sub-investigators<br>• Addition of a list of collaborators
				**Study synopsis and 4.1 Study entry criteria**<br>• Inclusion of cervical cancer stage I to III<br>• Exclusion criteria if previous intervention involvement.
				**4.3. Complex Intervention**<br>• In case of control arm contact to the Survivorship clinic, patients will be referred to their standard of care guidelines
				**4.4 Study procedures – Informed consent**<br>• Addition of a remote process if needed.
				**4.4 Study procedures – Nutritional assessments**<br>• Re-worded nutrition assessments
				**4.4 Study procedures – discontinuation criteria**<br>• Discontinuation criteria added.
				**4.6 Study Calendar – Intervention Arm**<br>• Delated EORTC QLQ – OV28<br>• Included better specified related to the instruments used for the study<br>• Description of the symptoms assessed with PROMIS and PRO-CTCAE during the study

CREC amend Ref	Document	Version	Date	Summary of Changes
				• Comorbidities assessment<br>• Updated dietetics tools:<br>**Removed:** Nutrition Interview<br>**Removed:** Nutrition Score (PGSGA)<br>**Added:** Diet intake assessments (multiple 24h diet recalls & FFQ<br>**Added:** Diet quality assessment (World Cancer Research Fund/American Institute for Cancer Research score)<br>**Added:** Diet Education<br>**Added:** Personalised nutrition counselling if identified at risk of malnutrition
				**4.6 Study Calendar – Control Arm**<br>• Delated EORTC QLQ – OV28<br>• Included better specified related to the instruments used for the study<br>• Comorbidities assessment<br>• Dietetic Tools<br>• **Added:** BIA measurement<br>• **Added:** Hand-grip strength<br>• **Added:** Diet intake assessments (multiple 24h diet recalls & FFQ<br>**Added:** Diet quality assessment (World Cancer Research Fund/American Institute for Cancer Research score
	Appendix 1a. Survey for Ongoing Monitoring of Symptoms	V2	8 Jan 2021	• Dietetic assessment: Updated Q22 (now Q21) on weight, appetite, and Q23 (now Q22) on physical activity<br>• Changes on PRO-CTCAE items:<br>- Swelling items<br>- Pain items: PRO-CTCAE library items for "General Pain", "Join Pain", "abdominal pain"<br>- "Altered bowel function- urgency" item has been eliminated<br>• Not PRO-CTCAE items, researchers developed items: "Fear of recurrence" and "location of pain and swelling"
	Appendix 1b. Triggers for visit to Clinic Visit and their Calculation	V2	8 Jan 2021	• Information about triggered symptoms in the study.
	Appendix 2a. Pre QOL survey	V2	8 Jan 2021	• Sociodemographic data has been modified.
	Appendix 2b. Post QOL Survey	V2	8 Jan 2021	• Sociodemographic data has been modified.
	*Appendix 3a. Dietetics PGSGA assessment	/	/	* Removed
	Appendix 3a. Dietetics Food Frequency Questionnaire	V2	16 Dec 2020	• Updated version of the instrument

CREC amend Ref	Document	Version	Date	Summary of Changes
	Appendix 3b. Dietetics Acceptance/ Non-acceptance criteria for clinic	V1	8 Jan 2021	• Information re dietetic clinic capacity
	Appendix 3c. Dietitian Clinic Record Form	V1	8 Jan 2021	• Dietitian CRF
	Appendix 5. Nurse Record Form	V2	8 Jan 2021	• It has been submitted a completely new version of the document, which include clinic information, medication, comorbidities, use of resources and dropouts.
	Appendix 6. Participant Information Leaflet-Informed Consent Form_Womens Malignancy Survivorship	V3	8 Jan 2021	• Inclusion of a statement of patient declaimer<br>• Erased the sentence "in which the blood samples and data have been obtained." As we are not collecting this information.<br>• Rewording data sharing
	Appendix 7. HCP Information and consent for interview-focus group	V2	8 Jan 2021	• Erased the sentence "in which the blood samples and data have been obtained." As we are not collecting this information.<br>• Rewording data sharing
**ECM 3 (fff) 09/03/2021**	Protocol	V5.0	16 Feb 2021	**Study Synopsis**<br>• Recruitment date modified.<br>• Clinic contact details included
				**Contacts – Sub-investigators**<br>• Name new dietitian<br>• Sub-investigator moved to collaborator<br>• New collaborator
				**Muscle Strength:**<br>• REMOVE: Jamar dynamometer (Lafayette Manual Muscle Testing (MMT) System 01165 Lafayette Instruments, Lafeyette, IN)<br>• REPLACE WITH: Jamar dynamometer (Jamar Hydraulic Hand Dynamometer, Model 091011725, Sammons Preston Roylan, Nottinghamshire, UK
				**4.4.7. Survivorship clinic**<br>• Information about resources given to patients.<br>• Information about the reminder process for the ePROs
				**Study Calendar – Intervention Arm**<br>• REMOVE: x's at 2months for diet intake assessments and diet quality assessments<br>• REPLACE: x's at 12months for diet intake assessments and diet quality assessments
	Appendix 3c. Dietitian Clinic Record Form	V2	16 Feb 2021	Minor changes on the information collected.

CREC amend Ref	Document	Version	Date	Summary of Changes
	Appendix 6. Participant Information Leaflet-Patients Informed Consent Form	V4	16 Feb 2021	Contact information from the clinic included.<br>Included the word patients in the name of the document.
	Appendix 7. Participant Information Leaflet-HCP Informed Consent Form	V3	16 Feb 2021	Contact information from the clinic included.<br>Changed name to keep consistency with the other PIL
	Appendix 8. GP letter	V1	16 Feb 2021	New document
	Appendix 9a. Care Plan Breast Cancer	V1	16 Feb 2021	New document
	Appendix 9b. Care Plan Gynae Cancer	V1	16 Feb 2021	New document
	Appendix 10a. Instructions ePRO Castor	V1	16 Feb 2021	New document
	Appendix 10b. Instructions T-Pro videocall	V1	16 Feb 2021	New document
	Appendix 11. Patient study recruitment advertisements	V1	16 Feb 2021	New document
	Appendix 12. Study patient diaries	V1	16 Feb 2021	New document
	Appendix 13a. Nurse-Dietitian Clinic Schedule&Instructions-Intervention	V1	16 Feb 2021	New document
	Appendix 13b. Nurse-Dietitian Clinic Schedule&Instructions-Control	V1	16 Feb 2021	New document
	Appendix 14a. Pathway Structure	V1	16 Feb 2021	New document
	Appendix 14b. Fatigue-Pathway	V1	16 Feb 2021	New document
	Appendix 14c. Hot Flashes-Pathway	V1	16 Feb 2021	New document
	Appendix 14d. FOR Pathway	V1	16 Feb 2021	New document
	Appendix 14e. Sleep Pathway	V1	16 Feb 2021	New document
	Appendix 14f. Vaginal Discomfort Pathway	V1	16 Feb 2021	New document

CREC amend Ref	Document	Version	Date	Summary of Changes
	Appendix 14g. Cognition Pathway	V1	16 Feb 2021	New document
	Appendix 14h. Emotional Distress Pathway	V1	16 Feb 2021	New document
	Appendix 14i. Joint Pain Pathway	V1	16 Feb 2021	New document
	Appendix 14j. Pain Pathway	V1	16 Feb 2021	New document
	Appendix 14k. Bowel Pathway	V1	16 Feb 2021	New document
	Appendix 14l. Urinary Pathway	V1	16 Feb 2021	New document
	Appendix 14m. Sexual Health Pathway	V1	16 Feb 2021	New document
	Appendix 14n. Swellling Pathway	V1	16 Feb 2021	New document
**ECM 3 (ww) 11/05/2021**	Protocol	V6.0	16 Apr 2021	**MAJOR CHANGES**<br>**Study synopsis & 4.1 Study entry criteria & 3.1. Design**<br>• Extension within 6 months of completion of primary curative therapy to within the 12 months. And high grade criteria for endometrial cancer patients dismissed.<br>**MINOR CHANGES**<br>**Study synopsis**<br>• Inclusion UCC study code<br>**2.3. Secondary Objectives**<br>• Reworded two secondary objective to be more specific.<br>**3.2. Anticipated Study timeline**<br>• Updated correct dates<br>**4.4.5 Instruments for data collection**<br>• The information has been reworded/changed format to be clearer on the instruments used.<br>• QALY typo errors.<br>• The PRO-CTCAE items won't have the skip option.<br>• Included more information about the nutritional assessment tools.<br>**4.4.6 Survivorship Clinic**<br>• Rewording in this section has been done to be clearer.<br>• Section 4.4.6 moved "nutritional assessment"<br>**6.0 Statistician Plan**<br>• Reworded the feasibility outcomes of the study.
	Appendix 8. GP letter	V2	16 Apr 2021	• Inclusion UCC study code

CREC amend Ref	Document	Version	Date	Summary of Changes
				• Extension within 6 months of completion of primary curative therapy to within the 12 months. And high grade criteria for endometrial cancer patients dismissed.
	Appendix 11. Patient study recruitment advertisements	V2	16 Apr 2021	• Inclusion UCC study code<br>• Extension within 6 months of completion of primary curative therapy to within the 12 months. And high grade criteria for endometrial cancer patients dismissed
	Appendix 2a Pre QOL Survey	V3	16 Apr 2021	• Rewording of socio-demographic questions<br>• Item 34 repeated (Item 4 form the EORTC QLQ – BR23). Amended with the correct item.<br>• (Only in appendix 2a): Appraisal of Self Agency-R likert-scale: there was missing an option "Totally agree"
	Appendix 2b Post QOL Survey	V3	16 Apr 2021	• Rewording of socio-demographic questions<br>• Item 34 repeated (Item 4 form the EORTC QLQ – BR23). Amended with the correct item.
ECM 3 (qqq) 19/10/2021	Protocol	V7	21 Sep 2021	**MAJOR CHANGES**<br>**4.1. Eligibility criteria**<br>• Broadening criteria.<br><br>**MINOR CHANGES**<br>**Front page**<br>• Study title changed to LYSA<br>• Reference numbers included<br>• Registry trial identification ClinicalTrials.gov added<br>**Study synopsis**<br>• New collaborator<br>**Inclusion criteria nº4**<br>• Clarification on the study criteria nº4: "On or recommended to comence endocrine therapy during the study period."<br>**4.4.10 Usability and Satisfaction**<br>• Rewording<br>**Appendixes (Renamed to "Study Documents version log)**<br>• Note included for clarification regarding the appendixes.<br>• Table included with version log of each document.
	App 13a. Nurse-Dietitian Clinic Schedule &Instructions-Intervention	V2	21 Sep 2021	• Dietitian baseline assessments days changed to avoid bias and promote data consistency.<br>• Change format to add clarity.
	App 13b. Nurse-Dietitian Clinic Schedule &Instructions-Control	V2	21 Sep 2021	• Dietitian baseline assessments days changed to avoid bias and promote data consistency.<br>• Change format to add clarity.
	App 4a. Usability and satisfaction survey	V2	21 Sep 2021	•Items included for a better approach of the end of the study process evaluation (note: this survey have not yet been used; they will start to be used on March 2022)
	App 4b. Interview schedule for feasibility process evaluation (for patients and HCPs)	V2	21 Sep 2021	•Items included for a better approach of the end of the study process evaluation (note: the interviews/focus groups will start to be done on March 2022)

CREC amend Ref	Document	Version	Date	Summary of Changes
ECM 3 (l) 05/04/2022	Protocol	V8	22 Feb 2022	• Site addition: University Hospital Galway<br>• Amend typo error in the Study Schedule: there is no Diet Education and Personalised nutritional counselling at the end of the study at the end of the study.<br>• Added Galway site lead as a sub investigator
Submission	Protocol	V9	16 Jan 2023	MINOR CHANGES<br>• The protocol has been updated to reflect the changes to the PIL ICF in (a) the "Summary of study documents versions" and (b) Section 13 "Study Documents Versions Log".

Study Synopsis			
**Brief Title**	The LYSA Trial		
**Title**	**Women's Cancer Survivorship: The LYSA (Linking You to Support and Advice) Trial**		
**Principal Project Reference**	WHI19CON	**Other project reference**	**UCC code:** 19137<br>**ICS code:** WHI19CON<br>**BCR code:** BCR-2019-09-ICS-UCC<br>**ClinicalTrials.gov Identifier:** NCT05035173
**Study Design**	Complex interventional study		
**Specific Aims**	To evaluate the feasibility of introducing a women's malignancy survivorship clinic incorporating symptom management through ePRO collection (complex intervention) into routine follow up care in patients with early-stage HR-positive breast and gynaecologic cancer post primary therapy		
**Sampling Method**	Randomized Control Design (Parallel Arms)		
**Sample Size**	200		
**Entry Criteria**	Women with early-stage breast/gynaecologic cancer within 12 months of completion of primary curative therapy:<br>*Breast cancer:* Stage I-III hormone receptor-positive (defined as estrogen receptor and/or progesterone receptor ≥ 1% and HER2-negative per ASCO-CAP guidelines on or recommended to commence adjuvant endocrine therapy during the study period.<br>*Cervical cancer:* Stage I to III treated with curative intent.<br>*Endometrial cancer:* treated with curative intent adjuvant radiotherapy +/- chemotherapy.		
**Intervention**	Women's Cancer Survivorship Clinic and ePRO/Symptom Management		
**Study Sponsor**	University College Cork (UCC)		
**Recruitment Date**	March 2021-March 2022		
**Primary Endpoint**	Feasibility of pilot clinic		
**Dissemination**	The results will be considered for submission to an appropriate scientific presentation and/or peer reviewed publication.		

<table>
<tr><td colspan="3">Contacts</td></tr>
<tr><td>PI</td><td colspan="2">Professor Roisin Connolly, MB BCh MD
Chair in Cancer Research, University College Cork
Director, Cancer Research @UCC
Western Gateway Building, 4.110
Western Road,
Cork, Ireland
T. 021 4922687; M: 087 3427341
E. roisin.connolly@ucc.ie</td></tr>
<tr><td>Co-PI</td><td colspan="2">Professor Josephine Hegarty
Head of the School of Nursing and Midwifery,
University College Cork
T. 021 4901462; M 087-4177062:
E. J.Hegarty@ucc.ie</td></tr>
<tr><td>Sub-Investigators</td><td colspan="2"></td></tr>
<tr><td>Statistician</td><td colspan="2"></td></tr>
<tr><td>Collaborators</td><td colspan="2"></td></tr>
<tr><td>Sites</td><td>HRB Clinical Research Facility
Cork University Hospital
Cork, Ireland

Contact details:
WHI Survivorship clinic at CUH
Cork University Hospital / University College of Cork
Phone: 0868223289
Email: CUH.WHI@hse.ie</td><td>HRB Clinical Research Facility
University Hospital Galway
Galway, Ireland

Contact details:
WHI Survivorship clinic at UHG
National University of Ireland Galway / University Hospital Galway</td></tr>
</table>

Table of content

1.0 Introduction ***16***

2.0 Study Hypothesis and Objectives ***18***

2.1 Hypothesis **18**

2.2. Primary Objective: **18**

2.3. Secondary Objectives: **18**

3.0 Study Plan ***19***

3.1 Design: **19**

3.2 Anticipated Study timeline: **19**

3.3 Primary Study Endpoint: **19**

4.0 Methods ***20***

4.1 Subject entry criteria **20**

4.1.1 Inclusion Criteria: 20

4.1.2 Exclusion Criteria: 20

4.2 Sample size justification **20**

4.3 Complex Intervention **21**

4.4 Study procedures **22**

4.4.1 Screening 22

4.4.2 Informed Consent 22

4.4.3 Randomisation Procedures 22

4.4.4 ePRO Enrollment and Procedures 23

4.4.5 Instruments for data collection 23

4.4.6 Survivorship Clinic (complex intervention) 28

4.4.8 Duration of the Intervention 31

4.4.9 Discontinuation of Subjects 31

4.4.10 Usability and Satisfaction 32

4.4.11 Data extracted from patient hospital records 32

5.0 Study Calendar ***33***

5.1 Intervention Arm **33**

5.2Control Arm **35**

6.0 Statistical Plan ***36***

7 .0 Safety and Adverse Events ***37***

7.1 Recording of Adverse Events **37**

8.0 Data Handling and Record Keeping ***37***

8.1 Confidentiality 37

8.2 Source Documentation 37

8.3 Case Report Forms 37

8.4 Records Retention 38

9.0 Ethical Considerations ***38***

10.0 Study Finances ***38***

10.1 Funding Source: 38

10.2 Indemnity for the performance of the study 38

11.0 Sponsorship ***38***

12.0. References ***39***

13.0. Study Documents Versions Log ***43***

1.0 Introduction

Cancer is recognised as one of the most prominent health care conditions worldwide with predictions of 21.7 million new cases (excluding non-melanoma skin cancer) by 2030, an increase from 14.1 million cases in 2012. Improvements in surveillance and treatments, has resulted in increasing numbers of individuals living with and beyond a cancer diagnosis (NCRI 2017).

With cancer survivor numbers increasing, optimising individuals' quality of life is a particular focus for the Irish health care system. The newly published National Cancer Strategy 2017-2026 has 52 recommendations (Government of Ireland 2017). Recommendation 41 is focused on the requirement to conduct a National Cancer Survivorship Needs Assessment to ascertain the most suitable model of survivorship health care for use in Ireland.

Patients have many and varied needs on the cancer survivorship trajectory. The health care system will be able to respond to these needs in a more coordinated way if a cancer survivorship pathway is formalised with particular emphasis being placed upon dealing with troublesome symptoms, supporting individuals to transition through the various stages of the cancer journey, encouraging the active participation of patients in care and helping individuals to live well with, through and beyond a cancer diagnosis.

This report notes that while survivorship care has been highlighted by health care professionals as a key target area to focus on in the Irish context, to date structured survivorship care pathways and symptom specific pathways, support systems appear to be underdeveloped. There is evidence that some centres have been engaged in developing centre specific survivorship programmes and pathways (National Coalition for Cancer Survivorship, 2018; Richards et al, 2014). On a national front the NCCP have promoted the 'Thrive to Survive' survivorship programme. The future direction of survivorship cancer care services in Ireland require survivorship to be recognised as a journey from diagnosis through cancer treatment and beyond. Cancer survivorship care requires the allocation and provision of appropriate resources in order to provide optimum results. The National Cancer Control Programme (NCCP) recommends the implementation of a survivorship pathway underpinned by the key survivorship principles of Assess, Link in, Link out and onward, Inform, Empower, Support & Services (ALLIES for cancer survivorship care) was recommended. In particular, patients should be offered one-to-one sessions with health care professionals at key transition points; a comprehensive discharge summary on treatment completion; access to cancer specific follow-up and survivorship specific clinics; engagement with a survivorship programme; easy and rapid access to care for the management of symptoms and issue resolution. The NCCP recommends that this pathway should incorporate community care support structures to aid in a shared care approach that can reduce acute service overload and reflect both individualised care and family involvement (Mullen and Hanan 2019).

This research seeks to address these recommendations by developing and pilot testing a cancer survivorship clinic at Cork University hospital.

The development of a Women's Cancer Survivorship Clinic within the HSE South/South West hospital group is a collaboration with the Enhancing Cancer Awareness and Survivorship Programmes (ECASP) at the School of Nursing at University College Cork; regional Cancer Support Services; Irish Cancer Society Services; amongst others. This multi-disciplinary nurse-led clinic will span the hospital system, community services and international survivorship efforts (Sidney Kimmel Comprehensive Cancer Center at Johns Hopkins, USA).

The proposed clinic strategically aligns with patient priorities. Its' goal is to identify and manage important symptoms experienced by women impacted by effects of cancer treatment. These are outlined in the National Cancer Strategy 2017-2026. Indeed, Professor Josephine Hegarty (co-applicant for this proposal) and her group provided the information supporting the NCCPs conduction of a national cancer survivorship needs assessment. Feedback from The Young Women with Breast Cancer Networking and Information Day held at the Cork Arc House (10-12-2019) further highlighted sexual health issues as a main priority, alongside management of menopausal symptoms, and optimal nutrition and exercise.

A collaboration with the Breast Cancer Survivorship team at Johns Hopkins, USA will incorporate knowledge sharing re educational materials for patients, annual survivorship conferences, symptom management pathways and experience with use of technology for electronic Patient Reported Outcome (ePRO) collection.

2.0 Study Hypothesis and Objectives

2.1 Hypothesis

The introduction of a women's malignancy survivorship clinic into routine follow up care will be feasible and will result in improved symptom management and quality of life in patients with early stage hormone receptor (HR)-positive breast cancer and gynaecologic cancer post primary curative therapy.

Should this intervention prove feasible, we plan to use the data generated from this feasibility study as the basis for a Quality Improvement (QI) initiative for our patients in the future, by offering this intervention to all of our patients as a standard component of clinical care.

2.2. Primary Objective:

- To evaluate the feasibility of introducing a women's malignancy survivorship clinic incorporating symptom management through ePRO collection (complex intervention) into routine follow up care in patients with early-stage HR-positive breast and gynaecologic cancer post primary therapy.

2.3. Secondary Objectives:

- To describe the symptoms experienced by patients with breast and gyneacologic cancers and evaluate the impact of the intervention on select reported symptoms over a 12-month period.
- To evaluate the impact of the survivorship clinic on quality of life using serial ePRO measures.
- To explore self-care agency and its relationship to quality of life and symptoms experienced in both arms.
- To evaluate adjuvant endocrine therapy medication adherence rates at 12 months after study entry in both arms, and factors associated with non-adherence in early-stage HR-positive breast cancer patients.
- To explore and assess the satisfaction of patient and Health Care Professionals with the intervention and to determine their perceptions of the systems usability.
- To estimate the resource use and economic impact of a women's malignancy survivorship clinic.
- To evaluate the impact of a dietetic intervention by assessing changes in dietary intake and diet quality over a 12-month period.
- To evaluate the impact of a dietetic intervention on body weight, lean body mass, fat mass, muscle strength and nutritional risk over a 12-month period.

3.0 Study Plan

3.1 Design:

Complex interventional clinical study.

This randomized controlled clinical trial (RCT) will be conducted to test both the 1) Feasibility of conducting a Women's Malignancy Survivorship Clinic in Ireland, in women with HR-positive breast cancer and gynaecologic cancer post primary curative therapy and 2) The hypothesis that female cancer survivors who participate in a survivorship clinic intervention incorporating ePRO collection and targeted symptom management are more likely to experience improvement in symptom burden and QOL compared to controls who did not receive partake in the targeted symptom management pathways.

Figure 1: Study Schema

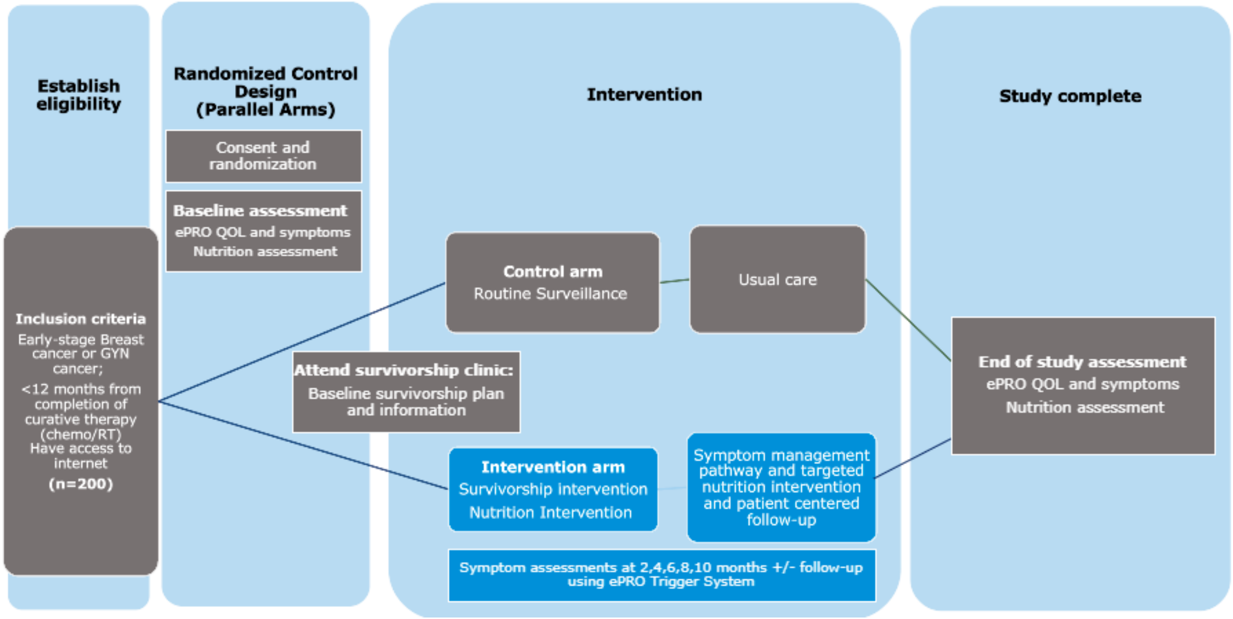

3.2 Anticipated Study timeline:

Subject recruitment: March 2021-March 2022

Subject follow up period: March 2021-March/April 2023 (12-13 months recruitment until 200 patients recruited + 12 months follow up for last patient enrolled)

Final data analysis: June 2023

3.3 Primary Study Endpoint:

Proportion of enrolled patients who complete the baseline and follow-up ePRO surveys.

4.0 Methods

4.1 Subject entry criteria

4.1.1 Inclusion Criteria:

1. Women aged > or = 18 years.
2. Ability to read and understand English.
3. Access to internet.
4. Early-stage breast/gynaecologic cancer within 12 months of completion of primary curative therapy:
   Breast cancer: Stage I-III hormone receptor-positive (defined as estrogen receptor and/or progesterone receptor ≥ 1%) and HER2-negative per ASCO-CAP guidelines on or recommended to comence adjuvant endocrine therapy during the study period (Allison, Hammond, Dowsett et al., 2020; Wolff, Hammond, Allison et al., 2018).
   Cervical cancer: Stage I to III treated with curative intent.
   Endometrial cancer: treated with curative intent adjuvant radiotherapy +/- chemotherapy.

4.1.2 Exclusion Criteria:

1. Patients who are not treated with curative intent as above.
2. Patients with premalignant disease (e.g. DCIS/LCIS).
3. Persons who, in the opinion of the researcher or supervising clinician, are unable to cooperate adequately with the study protocol.
4. Recent (within 12 months) participation in a study/programme involving a lifestyle intervention (e.g diet, exercise, survivorship). Note: Per discretion of PI as to whether may impact the outcome of this study intervention.

4.2 Sample size justification

Given that the main objectives of this study are to evaluate the *feasibility* of introducing a women's malignancy survivorship clinic, we have not calculated the sample size that would be required to reliably estimate the effect of the clinic on patient outcomes. This follows from established best practices in pilot and feasibility studies (Whitehead, Sully, & Campbell, 2014) for which there is usually too much uncertainty in the various factors that are needed to make a sample size calculation robust (e.g. effect sizes of interest, outcome variability, etc.). Our sample size justification is instead based on trying to recruit the largest possible sample given existing financial and pragmatic constraints. This will allow us to maximize our ability to identify barriers to implementation, as well as the information that will be required to properly design any subsequent trials of the clinic's efficacy (e.g. robust estimation of estimator variability, even for binary outcomes (Teare, Dimairo, Shepard et al., 2014). At the same time, the potential costs of over-recruitment are largely absent for this study. Most importantly, there are no excess risks to patients receiving the intervention, and all patients are guaranteed access to the current standard of care. Further, most of the financial costs of the study are fixed, regardless of the actual number of

patients recruited into the study. With these points in mind, based on the current numbers of patients coming through existing services, we anticipate recruiting approximately 140 patients with early stage breast cancer from the Orchid Centre in Cork University Hospital over the first 12 months of the study (approximately 3 patients per week). We further anticipate being able to recruit 60 patients with cervix/endometrial cancer over the 12 month period (approximately 1 each week). This will amount to approximately 200 patients, which will leave us in an excellent position to meet the objectives of the study (Viechtbauer, Smits, Kotz, et al., 2015; Whitehead, Julious, Cooper & Campbell, 2016).

4.3 Complex Intervention

The Women's Cancer Survivorship Clinic (complex intervention) within the HSE South/South West hospital group is a collaboration with the Enhancing Cancer Awareness and Survivorship Programmes (ECASP) at the School of Nursing at University College Cork; regional Cancer Support Services; Irish Cancer Society Services; amongst others.

This multi-disciplinary nurse-led clinic will span the hospital system, community services and international survivorship efforts (Sidney Kimmel Comprehensive Cancer Center at Johns Hopkins, USA). Participation in the study (intervention arm) will include:

1. Informed consent and ePRO enrollment
2. Baseline questionnaire/needs assessment
3. Receipt of Survivorship Plan
4. Education and Symptom Management Plan
5. Dietetic Evaluation
6. Follow up questionnaire/needs assessment

Symptom pathways will be available for common symptoms assessed in the ePRO measures such as joint pain, depression, anxiety, vaginal dryness, dyspareunia, hot flashes, cognitive impairment and fatigue.

Those in the **intervention arm** will undertake ePRO at baseline, 2, 4, 6, 8, 10, 12 months. Triggers to the study team will prompt symptom evaluation and management in between routine clinic visit. A phone call from the clinic nurse will take place @ 6 months also to follow up on progress, symptoms etc.

Those in the **control arm** will undertake ePRO at baseline12 months. They will have usual follow up care over the 12 month period of the study, after attendance at the Survivorship Clinic. Where participants in the control arm contact the Survivorship team for clinical or other advice, they will be referred to the usual care pathway.

Patient stakeholders/advocates will help to co-design the symptom management resources and the survivorship clinic in partnership with the project team. They will be an integral part of the Steering Committee which will lead the development and conduct of the clinical and research components of the pilot until completion.

The Clinical Nurse Specialist will work alongside clinic staff at the Orchid Centre; supervised by consultants and supported by a research assistant. This latter position will involve development of educational materials, promotional material, educational events, data collection etc. A post-doctoral researcher will coordinate research data collection and analysis under the supervision of Professors Connolly and Hegarty and the wider research team. Further information related to the complex intervention can be found in section next Study Procedures section under the heading "4.4.6. Survivorship Clinic (Complex intervention)".

4.4 Study procedures

4.4.1 Screening

Referrals to the clinic will be via the Breast/GYN multi-disciplinary meetings, the medical/surgical and radiation oncology clinics and direct from our Clinical Nurse Specialists and GP practices. In reality, the majority of patients will be enrolled at their first post-operative visit with the medical oncologist. The study team will thus capture eligible patients after completion of primary therapy for their cancer, who have entered a surveillance schedule.

4.4.2 Informed Consent

Patients will be consented by a member of the study team. This may occur in person, as they present for their routine clinic visit. Alternatively, this may involve a remote consenting process (e.g pending geographic or COVID-related barriers as follows:

- The potential participant will receive a hard copy of the PIL/ICF by email or post.
- A member of the study team will telephone (or may use skype, zoom etc.) the potential participant, and go through the PIL and answer any questions which participant may have
- If the patient is happy to take part the researcher will ask them to sign and date the ICF and send it back to the study team by email (scan the document or take photograph and send as email attachment) or by post.
- The member of the study who explained the study to the patient will then sign and date the ICF.
- The completed ICF (now signed and dated by both participant and research team member) will be filed in the study file which is kept securely. One copy will be placed in the patients medical notes and one copy will be emailed or posted back to the participant for their own records.
- Once the signed ICF has been filed in the study file it will be detailed from email system.

4.4.3 Randomisation Procedures

Randomization with be stratified by care pathway, i.e. there will be one randomization list for breast cancer patients, and one for cervical/endometrial cancer patients. Randomization within strata will then be restricted using randomly sized blocks of size 4, 6 or 8. The randomization lists will be generated by the electronic data management system. Further, any given allocation will be

concealed from the research team until that patient is unambiguously consented and enrolled onto the trial.

4.4.4 ePRO Enrollment and Procedures

ePROs will be collected using an electronic data capture (EDC) system. This service is offered through the Clinical Research Facility-Cork (CRF-C), and meets national and international guidelines with respect to data privacy and protection standards. CRF-C staff will support the development of the ePRO survey template, study database construction and provide investigator training for downstream data collection, monitoring and analyses. Patient names will not be sent to the EDC database.

Patients will be emailed a link to the ePRO Symptom Survey at two-monthly intervals over the course of a 12 month period. Surveys can be accessed through any standard internet browser. Responses will be immediately uploaded to the study EDC database. Notification of severe or worsening ePRO scores will be calculated and notified to the Nurse in Charge of the Clinic. The severe or worsening ePRO symptoms scores will follow a trigger system which can be consulted in appendix 1b.

4.4.5 Instruments for data collection

We seek to survey the effect of the intervention on key ePROs, i.e. health related quality of life (HRQOL) and symptoms experienced. Generally, HRQOL covers the subjective perceptions of the positive and negative aspects of cancer patients' symptoms, including physical, emotional, social, and cognitive functions and, importantly, disease symptoms and side effects of treatment.
Information collected via ePROs surveys using EDC:

- **The ePRO Quality of Life Survey will be administered Pre and Post the intervention** (baseline and end of the study both arms). Paper version available in appendix 2a (pre) and 2b (post).
- **The ePRO Symptom Survey Instrument is for ongoing monitoring of symptoms and concerns** (baseline, month 2-4-6-8-10 and end of the study intervention arm; baseline and end of study control arm). Paper version available in appendix 1a. All the items in this survey include a trigger; the trigger system can be consulted in appendix 1b.

The ePRO Quality of Life Survey (administered Pre and Post the intervention)

The Pre and post intervention survey is comprised of Quality of Life measure (The European Organisation for Research and Treatment of Cancer Quality of Life Questionnaire Core 30 (EORTC-QLQ-C30)); Quality-adjusted life-year (QALY) (EQ-5D-5L) and a measure of self- care agency/power to self-care. We seek to determine the effectiveness of the intervention on Quality of Life (core quality of life of cancer patients and disease). The Quality of Life Survey ePRO package include the following instruments:

A) The European Organisation for Research and Treatment of Cancer EORTC QLQ-C30 comprises 30 items that can be summarized in 15 scales: Physical Functioning (PF),

Role Functioning (RF), Social Functioning (SF), Emotional Functioning (EF), Cognitive Functioning (CF), Global QOL (QL), Fatigue (FA), Pain (PA), Nausea/Vomiting (NV), Appetite Loss (AP), Dyspnea (DY), Sleep Disturbances (SL), Diarrhoea (DI), Constipation (CO), and Financial Impact of Disease (FI). The European Organisation for Research and Treatment of Cancer (EORTC) QLQ-C30 is a patient-reported outcome measure to assess HRQoL among cancer patients (Mierzynska t al, 2020). The disease specific HRQOL will be collected using EORTC modules for breast, endometrial and cervical cancer as required. These modules have undergone all of the EORTC phases of development (1–4), robust international psychometric testing and rigorous translations according to the EORTC standards.

B) QALY is a generic measure of disease burden, including both the quality and the quantity of life lived. It is used in economic evaluation to assess the value of healthcare interventions. For the economic evaluation, HRQoL is assessed using the EQ-5D-5L (Buchholz et al, 2018; Kouwenberg et al 2019). The EQ-5D-5L will be administered at T0, T12. The EQ-5D-5L consists of five questions evaluating the following health dimensions: mobility, self-care, usual activities, pain/discomfort, and anxiety/depression. The patients' EQ-5D-5L health states will be transformed into utility scores.***C) Appraisal Self-Care Agency****:* The ASAS-R is used to measure self-care agency. The 15-item tool has three subscales Having power for self-care; Developing power for self-care and Lacking power for self-care (Sousa et al, 2010).

ePRO Symptom Survey Instrument for ongoing monitoring of symptoms and concerns.

The survey tool was developed based upon a review of literature, consultation with key stakeholders including clinicians and patients. The team sought to balance the burden of completing lengthy survey tools with collecting data on the frequency, severity and perceived interference of symptoms usually reported by patients with breast and gynaecological cancer. A core list of symptoms/ adverse events were identified including gastrointestinal (constipation, diarrhoea, faecal incontinence, altered bowel function- urgency), attention/memory, pain (aching joints, abdominal pain, general pain), fatigue and insomnia, mood-emotional distress/depression, anxiety, fear of cancer recurrence), gynaecological (vaginal dryness), urinary (urinary urgency, urinary frequency, change in colour of urine, urinary incontinence), sexual (decreased libido, vaginal dryness, female vaginal discomfort), diet (weight loss, reduced appetite; concern about weight gain), exercise and other areas (hot flashes). Further patient feedback on survey items will be sought prior to commencing the pilot study. The Ongoing Monitoring Symptom Survey ePRO package include the following instruments:

A) **The Patient-Reported Outcomes version of the Common Terminology Criteria for Adverse Events (PRO-CTCAE)** (Kluetz, Chingos, Basch et al., 2016). Items measuring frequency, severity, and interference with daily activities used a 0–4 rating scale (i.e. Frequency item: How often did you have [named symptom]? (0) never, (1) rarely, (2) occasionally, (3) frequently, (4) almost constantly; Severity item: What was the severity of

your [named symptom] at its worst?: (0) none, (1) mild, (2) moderate, (3) severe, (4) very severe); Interference item: How much did [named symptom] interfere with your usual or daily activities?: (0) not at all, (1) a little bit, (2) somewhat, (3) quite a bit, (4) very much). The response options for presence/absence were (0) no or (1) yes. The standard recall period for all PRO-CTCAE items is the past 7 days. Response options are scored 0-4. Higher scores indicate higher frequency, greater severity and more interference.

B) **The Patient-Reported Outcomes Measurement Information System (PROMIS)** (Cella, Choi, Garcia et al 2014; Cessna, Jim, Sutton et al 2016). The symptoms measured with this instruments are Fatigue (V1.0 - Short Form 4a), Anxiety (V1.0 - Short Form 4a) Depression (1.0 - Short Form 4a) and Vaginal Discomfort (V1.0 - Female Vaginal Discomfort). Fatigue, Anxiety and Depression questionnaires consist of three subdomains that include 4 questions each. Patients chose responses from a Likert-type scale consisting of five points: (never, rarely, sometimes, usually, and always). Each 4 item subscale is scored separately, with a total score range of 4 to 20, with higher scores indicating higher levels of distress or fatigue. Vaginal Discomfort questionnaire consist of 3 questions. Patients chose responses from a Likert-type scale consisting of 4 points (have not had any sexual activity in the past 30 days, very comfortable, comfortable, uncomfortable, and very uncomfortable) and 2 Likert-type scale consisting of 5 points (never, rarely, sometimes, usually, always). These measures are scored by transforming raw scores to T scores that are normed on a sample that matched the U.S. general population with respect to age, sex, race/ethnicity, and education. Tables are used to translate the total raw score into a T-score for each patient. T scores have a mean of 50 and standard deviation of 10. A higher PROMIS T-score represents more of the concept being measured. For positively-worded concepts like Emotional Support, a T-score of 60 is one SD worse than average. By comparison, an Emotional Support T-score of 40 is one SD better than average. PROMIS measures are calibrated using an item response theory (IRT) model. For each item, there is a single, most likely response associated with each possible PROMIS T score. For example, the response for the item, "My sleep was restless" is most likely to be "not at all" for individuals with low sleep disturbance and "very much" for individuals with high sleep disturbance.

C) **Malnutrition Screening Tool:** The MST is a simple, quick, valid and reliable tool which can be used to identify patients at risk of malnutrition. It asks participants if they have lost weight recently without trying (and if so, how much) and if they have been eating poorly because of a decreased appetite (Ferguson et al. 1999). Patients identified at nutritional risk in the intervention arm only, will be advised on an appropriate nutrition care plan. Some patients may require ongoing monitoring, nutritional counselling and review of personal nutrition care plans. These patients will be invited to attend the ongoing dietitian clinic. The need for follow up review and number of sessions required for each patient will be decided by the clinic dietitian based on clinical judgment. In the case where the dietetic caseload may exceed the capacity of the survivorship clinic, an acceptance and

non-acceptance criteria for the dietetic clinic caseload was developed and will be used if required (Appendix 3b). These criteria will ensure that patients requiring more intensive and/or specialised dietetic support will be offered appropriate dietetic care in a timely manner.

D) **Physical activity:** a short validated physical activity questionnaire will be used (Danquah et al 2018) to measure physical activity levels. Physical activity is a fundamental Cancer Prevention Recommendation of the World Cancer Research Fund/American Institute for Cancer Research (WCRF/AICR) framework for cancer prevention. It will be measured as moderate to vigorous physical activity in minutes per week. It will be included in the (WCRF/AICR) standardised scoring system (Shams-White et al 2019). This is a practical tool that operationalises eight out of ten 2018 WCRF/AICR cancer prevention recommendations. The score enables researchers to assess the degree to which people adhere to the Cancer Prevention Recommendations and the health effects of doing so. The WCRF/AICR score is an accumulation of points assigned for adhering – or not adhering - to each of the components of the Cancer Prevention Recommendations or sub-recommendations. However, physical activity won't be included in the triggers.

Information collected via other sources:

Nutritional assessments

Both arms of the study will partake in nutritional assessments at baseline and the clinic endpoint (12 months). Evaluations will include assessment of dietary intake and dietary quality. Body weight and muscle strength will be measured. Body composition will be analysed.

Dietary intake: *D*ietary assessments will be conducted by a dietitian. This includes two 24 Hour Dietary Recalls and a Food Frequency Questionnaire (Appendix 3) t at baseline and the clinic endpoint (12 months). Portion sizes will be validated through the use of a food atlas.

- **24 Hour Dietary Recall:** Following assessment of the 24h diet recalls, the clinic dietitian will assign appropriate food codes to each item reported. Data analysis will be limited to the McCance and Widdowson's composition of foods integrated dataset, the USDA Food Composition Databases and the Irish food composition databases. Food coding will be completed following each individual dietetic assessment. A food code data base will be generated and expanded upon following extraction of new food codes from each new 24hour diet recall recorded. This data will be analysed using Nutritics, web-based nutritional analysis software to capture diet intake assessing macro- and micronutrients, and specific food and nutrients directly relating to the dietary quality outlined in the WCRF framework for cancer prevention. All reference data will be assessed within-individual comparison and between-individual comparison. All data will be exported from Nutritics into a Microsoft excel file for processing with the study statistician.

- **Food Frequency Questionnaire:** Individual and collective sample data captured from the EPIC-Norfolk Food Frequency Question (FFQ) will be entered in to the FFQ processing tool, FETA (Φετα) FFQ EPIC Tool for Analysis, which is based on the earlier CAFÉ system (Welch et al. 2005). The FETA tool is free to use and will produce different levels of nutrient data, as well as basic food groups and food patterns. This programme is based on version 6 (CAMB/PQ/6/1205) of the EPIC-Norfolk FFQ. FETA calculates the average daily intake of 46 nutrients and 14 food groups, for each individual. The default nutrients list provides a description of each nutrient/food group and the units used. The nutrient data for the FFQ foods have come from McCance and Widdowson's "The Composition of Foods (5th edition)" and its associated supplements. In FETA, the frequency category is converted into a portion multiplier (e.g. once a week = 1/7 = 0.14). After multiplication with the portion size, an average daily food weight for each of the 130 FFQ items is obtained. These weights are multiplied by the nutrient composition per gram to obtain the nutrient composition of the actual amount eaten. After summing all FFQ items for a participant, an average daily nutrient intake is obtained. Individuals with more than 10 missing lines of data will be excluded. The top and bottom 0.5% of the ratio of energy intake to estimated basal metabolic rate will be flagged as extreme outliers of nutrient intakes. All data will be exported from FETA data processing report into a readable Microsoft excel file for processing by the study statistician.

Information collected via the study database in the EDC:

Data related to patient comorbidities, medication, and clinician record of actions/follow-up at survivorship and symptom management encounters: Data relating to resource utilisation will be collected on an ongoing basis for the intervention group and at the end of the study for the control group. Data relating to survivorship services that were provided by the CUH and used during the 12 month period will be extracted from the patient records. Data on service utilisation (outside of CUH) will be collected at T0 and T12 by the information provided from the patient through the study patient's diaries (appendix 12); this will include the services used, frequency of use, duration of each service and the personnel responsible for providing the service. This information will be collected by the Research Nurse.

Nutritional assessments:

Dietary quality will be measured using the WCRF/AICR standardised scoring system which was discussed earlier. This also includes assessment of their alcohol intake, whether mothers' breastfed their babies and for how long.

Anthropometric measures:

- **Body weight:** Weight will be recorded with the participant wearing light clothes and no shoes on the same scale at the baseline clinic visit and at the clinic endpoint (12 months). Additionally, the intervention arm will also self-assess their weight

using the same personal scales at home, at the same time of the day, at baseline and at 2 monthly intervals until and including the endpoint of the study. Participants in the intervention arm will report their weight on the same day their weight has been recorded at home through their two monthly ePRO surveys.

- **Height:** The participant's height will also be measured at baseline in clinic.
- **Body Mass Index (BMI):** This will be calculated using the participant's weight and height and is part of the WCRF/AICR scoring system.
- **Body Composition:** Segmental body composition will be analysed using Bioelectrical Impedence Analysis (BIA) with the Body Composition Monitor (BCM) (Fresenius Medical Care, Bad Homburg, Germany). This is used for an accurate and objective assessment of overall body composition and hydration status (Baumgartner et al, 1988). The BCM device calculates the LBM, lean tissue index (LTI), fat tissue index (FTI), body cell mass (BCM), and adipose tissue mass (ATM), extracellular (ECW), intracellular (ICW) and total body water (TBW) based on measurement values, using physiological modelling and mixture equations (Cole-Cole plot and Hanai formulae andChamney et al, 2007).
- **Waist circumference:** This measurement will be recorded with the participant not wearing clothing that is directly over the skin. If this is not possible, the measurement may be taken over light clothing. This is also part of the WCRF/AICR standardised scoring system.
- **Muscle strength:** Hand grip strength (HGS) will be measured using the Jamar dynamometer (Jamar Hydraulic Hand Dynamometer, Model 091011725, Sammons Preston Roylan, Nottinghamshire, UK) with its handle in the second position as recommended by the American Society of Hand Therapists (ASHT)(Fess,1986). Grip strength will be measured 3 times per hand, alternating between left and right hand to allow rest and account for any fatigue.
- **Nutritional risk:** Participant's nutritional status will be monitored over the 12 month period on the study database by assessing any change in their Body Composition and MST score.

4.4.6 Survivorship Clinic (complex intervention)

Research and relevant Clinical Staff will be trained in the protocol. Following successful completion of the Ethics Committee approved informed consent process, a unique study identifier will be applied to all subject's study data and will be used exclusively as a unique identifier in study Case Report Forms. Subject's clinical notes will be reviewed, and an appointment arranged at a mutually convenient time to attend the Orchid Centre clinic for the baseline Survivorship clinic visit:

- **For the intervention arm**, the initial Survivorship Clinic visit in the Orchid Centre clinic at CUH will outline a Survivorship Care Plan by the Nurse (Care Plan; see appendix 9a

and 9b), assess and manage current symptomatology and needs per management pathways, refer to appropriate specialists, and ensure future access to the clinic where needed during the 12 month period (see Study Schema Figure 1). Patients in this arm will receive Education and Symptom Management Plan, depending on the outcome of ePRO Symptoms Surveys, or according to clinical judgement (for further information see appendix 14a). The physical nutritional assessments will be undertaken and required Diet Education and Personalised Nutrition Counselling will be performed (For further information see Nutritional Intervention section).

- **For the control arm**, the baseline Survivorship Clinic visit in the Orchid Centre clinic at CUH will outline a Survivorship Care Plan by the Nurse (Care Plan; see appendix 9a and 9b). These patients will follow their usual standard of care outside of the baseline and end of study survivorship clinic visits (see Study Schema Figure 1). The physical nutritional assessments will be undertaken.

At the baseline clinical visit both arms may be advised to use publicly accessible resources currently used in standard clinical care including, but not limited, to the following:

- CUH Exercise Booklet
- ICS Survivorship Booklet or specific Irish Cancer Society Symptom booklets/leaflets
- Irish Cancer Prevention Network Leaflet
- UCC Healthy Eating for Cancer Survivorship
- HSE Let's Get Active leaflet
- Get Ireland Walking Sample Walking Programmes
- HSE Alcohol and Health Leaflet
- Online Websites: ICS, Macmillan.org.uk, Marie Keating, Breast Cancer Ireland, Daisy Network, ARC house etc...
- APPs: Calm, iBreath, Headspace

At the end of the study, both arms will have an appointment, arranged at a mutually convenient time to attend the Orchid Centre clinic for the end of the study Survivorship clinic visit. The physical nutritional assessment will be reassessed at this point.

Follow up Assessment and ePRO Trigger System for the intervention group

Patients will complete ePRO questionnaires per Study Calendar depending on which arm of the study they are enrolled in. In addition, patients will self-report weight, and current adjuvant endocrine therapy adherence at each time point.

The Study Team will be aware of severe or worsening ePRO scores and pointed to brief, evidence-based Symptom Management Pathways that outline recommended interventions for managing the relevant symptoms. These evidence-based pathways are based on up to date standard recommendations in oncology clinical guidelines such as those provided by NCCN and ASCO (Study-developed evidence-based pathways for each symptom included in the study are available from appendix 14a to 14n; appendix 14a corresponds to the study pathway structure). We have

pre-defined score thresholds beyond which each symptom will be considered severe enough to trigger an alert. In addition, a worsening of score compared to baseline exceeding a pre-defined threshold will trigger an alert (Further information about study triggers are available in appendix 1b; and information about how a trigger is assessed by the CNMII is available in appendix 14a).

We have selected pre-defined thresholds to identify severe or worsening symptoms based on scores on the ePRO measures and will monitor these thresholds on an ongoing basis. For each ePRO measure, thresholds will be selected to identify severe symptoms or clinically significant worsening of symptoms based on recommended scoring thresholds and prior use of each questionnaire. The selected thresholds to trigger alerts will be reviewed by patient and provider focus groups and will be modified if necessary. Proposed thresholds to trigger an alert for each of the proposed questionnaires are attached as Appendix 1b. Score alerts for PRO-CTCAE measures will be triggered for scores in the severe/very severe categories and for scores that worsen by at least 2 points compared to baseline on a 5 point scale. For the PROMIS measures other than sexual function, score alerts will be triggered for T scores more than 2 standard deviations more severe than the population mean score or by T scores that worsen by more than 0.5 standard deviations compared to baseline. The alert trigger for the PROMIS sexual function scale will be for T scores more than 1.5 times more severe than the population mean or that worsen by more than 0.5 standard deviations compared to baseline. These thresholds were selected based on review of the available literature regarding these questionnaires and are thought to identify severe symptoms and/or clinically significant change in symptoms. Trigger points can be overridden by clinical judgment if required and such action will be documented in the Nurse Record Form.

Since PROMIS T scores cannot be calculated if a patient does not complete all questions in the questionnaire and we do not want to miss clinically significant symptoms of depression or anxiety, an alert will also be triggered in the setting of incomplete depression and anxiety questionnaires if the answer to any key individual question is “often” or “always”.

When addressing the symptoms identified through the ePRO measures, the first step in each recommended clinical pathway is to confirm the presence of the symptom(s). By doing so, we will be able to refine the trigger thresholds and to ensure that the pathways are followed when clinically appropriate. We will record scores that have been triggered and track clinical interventions in response to ePRO score alerts in the patient medical chart and extract the data for analyses by chart review.

Patients will be given at baseline the dates when they will be receiving the surveys. Patients will be reminded twice to complete the surveys as follows:

- 1st reminder: an automatic reminder will be set up if the system does not detect the survey completed in 24h.
- 2nd reminder: a research team member will call the patient if the survey is not completed with 72h.

After a period of 7 days, the survey will be locked, and the patient will no longer be able to answer that survey.

Nutrition for the intervention arm

The nutrition intervention will include a personalised approach to nutrition education for patients in the intervention arm only. Patients will complete a symptom survey at 2 monthly intervals throughout the study. The symptom survey will act as screening tool to identify nutritional risk. Patient specific nutrition counselling will be offered to patients identified at risk.

Diet Education and Personalised Nutrition Counselling: The main goal of the nutritional intervention is to improve the diet quality of each patient using a standardized nutrition assessment, offering evidence-based diet education and developing personalized nutrition goals. Individual goals and targets will be set during the 1st consultation. Dietary counselling involves the prescription of a therapeutic diet that uses regular foods, which can be further modified to provide for individual requirements. The therapeutic diet is adjusted to the individual's usual diet, thereby recognizing personal eating patterns and preferences, which form the basis for individualized dietary counselling. The prescription identifies the type, amount, and frequency of feeding and specifies the caloric/protein level to attain, together with any restrictions and limited or increased individual dietary components. In addition, nutritional advice can be adapted to respond to specific nutrition impact on symptoms (e.g. dysphagia, constipation, diarrhoea, altered taste and temperature sensitivity). Energy and protein requirements will be calculated only for patients that require specific caloric and protein targets to meet their individualised nutritional goals.

During the initial consultation every patient will be educated on diet and lifestyle factors associated with cancer prevention, based on the World Cancer Research Fund's recommendations (WCRF, 2018). After the initial nutrition assessment, dietetic follow up will be individualised. This includes the number of dietetic sessions required for each patient. This will be decided at the dietitian's discretion based on clinical judgement. In the case where the dietetic caseload may exceed the capacity of the survivorship clinic, an acceptance and non-acceptance criteria for the dietetic clinic caseload was developed, and will be used if required (Appendix 3b). These criteria will ensure that patients requiring more intensive and/or specialised dietetic support will be offered appropriate dietetic care.

4.4.8 Duration of the Intervention

Patients in the intervention group will participate in the intervention for up to 12 months. They will complete surveys during the first 12 months of the study at 2 month intervals. Charts will be reviewed up to 4 weeks after the last survey to document clinician interventions in response to alerts.

4.4.9 Discontinuation of Subjects

All patients who initiate the study will be included in the overall analysis. All reasons for discontinuation of participation in the trial will be documented clearly in the trial record.

The reasons for discontinuation may include:

a) At subject's own request.
   Note: The reason for discontinuation from the study must be documented.
b) Death.
c) Intercurrent illness or condition that would, in the judgment of the principal investigator, affect assessment of clinical status to a significant degree.
d) Evidence of disease recurrence during study.
e) Study is terminated for any reason.
f) Subject withdraws consent for follow-up.
g) Subject does not complete 2 consecutive ePRO surveys. In that case, the patient will be given the option to continue receiving surveys or to withdraw from the study.

4.4.10 Usability and Satisfaction

In order to gather information on usability and satisfaction with the computerized questionnaire system, the opinions of patients will be gathered using a modified five-item Usability Survey and four item Satisfaction Survey (Lewis, 1995 and Sharma et al, 2016). The questions in this index are designed to assess different domains of usability of the web-based system, including usefulness, ease of use and satisfaction with use, and were adapted from a previously developed computer usability and satisfaction questionnaire. The survey will also include some open-ended questions allowing participants to share their perspectives more freely.

As part of the process evaluation, all patients, health care professionals and the broader team involved in the development and implementation of the clinic will be invited to attend either a focus group or one to one interview that will take place after the completion of their involvement in the study (in person or remotely). Attendees will be asked about their experiences of the survivorship clinic and associated processes, limitations, challenges and possible benefits and future possibilities concerning the intervention. The interview will be audio-recorded with the consent of participants (both patients and HCPs), and will last approximately one hour. Transcribed audio will be analysed using qualitative content analysis. A priori it is anticipated that we will interview up to twenty patients, ten members of the MDT and ten broader team/stakeholders/service providers. A lesser number may be required if data saturation is attained earlier.

4.4.11 Data extracted from patient hospital records

Patient data including age, sex, cancer site, diagnosis, treatment, current medications will be extracted from the patient records exclusive of patient identifiers from The Orchid Centre database located in Cork University Hospital using the Nurse Record Form. All survivorship services that were provided by the CUH and used during the 12 month period will be extracted from the patient records.

5.0 Study Calendar

5.1 Intervention Arm

Actions and Instruments[b]	Baseline[a] (Months) →	2	4	6	8	10	12
**Information provided; recruit to study; consent form; GDPR Data form, visit to survivorship clinic, meet dietician- dietetics assessment (e.g. BMI, diet)<br>Baseline: patient clinical and socio-demographics collected**	X						
**Randomisation**	X						
**Current medications**	X	X	X	X	X	X	X
**Comorbidities**	X	X	X	X	X	X	X
**ePRO Health related-Quality of Life survey. Instruments used as follows:<br>1. EORTC-QLQ-C30 (30 items)<br>2. *Considering patients cancer type:*<br>• Cervical cancer: EORTC QLQ – CX24 (24 items)<br>• Endometrial cancer: EORTC QLQ – EN24 (24 items)<br>• Breast cancer: EORTC QLQ - BR23 (23 items)<br>3. EQ-5D-5L (5 items) (measures QALY)<br>4. Appraisal Self-Care Agency-R (15 items) (measures power to self-care)**	X						X
**ePRO Patient-reported symptom questionnaire includes PROMIS and PRO-CTCAE items<br>Fear of cancer recurrence, Adherence**	X	X	X	X	X	X	X
**Body weight**	X	x	x	x	x	x	X
**BIA measurement**	X						X
**Hand grip strength**	X						X
**Diet intake assessments (multiple 24hour dietary recalls & food frequency questionnaire)**	x						X
**Diet quality assessment<br>(World Cancer Research Fund / American Institute for Cancer research score)**	x						X
**World Cancer Research Fund/American Institute for Cancer Research (WCRF/AICR) score**	X						X
**Diet Education**	x						
**Personalised nutrition counselling if identified at nutritional risk[c]**	x						
**Use of resources form**				X			X
**Follow up phone call by clinic nurse**				X			
**End of study patient questionnaire—includes system usability, satisfaction scales**							X
**Qualitative interviews with patients, healthcare professionals and wider team, stakeholders (process evaluation)**							X
**Clinician record of actions/follow-up at survivorship and symptom management encounters<br>Data extracted from hospital records**		X	X	X	X	X	X
**Drop out date, reason (data collected as required)**							

a. Baseline measures may be completed up to 2 weeks after enrollment and prior to attendance at the initial clinic visit. For patients who do not complete the baseline measures, the date for the follow-up measures will be determined using the enrollment date (i.e. 2, 4, 6, 8, 10, 12 months from the date of enrollment)

b. The every 2 month measures may be completed up to two weeks after each survey becomes available. Electronic survey reminders will be sent to patients to facilitate ePRO completion. Dates for all follow-up measures will be based on the enrollment date.
c. Repeat dietetic collections if referred to dietitian from diet trigger questions and/or if remain on dietitian caseload from baseline interview. This will be based on individual needs.

Note: Clinical follow-up will be according to standard of care. Follow-up visits are not specifically required per protocol other than those needed for routine clinical care.

5.2Control Arm

Actions and Instruments	Baseline[a]	12 Months
Information provided; recruit to study; consent form; GDPR Data form, visit to survivorship clinic, meet dietician- dietetics assessment (e.g. BMI, diet)<br>Baseline: patient clinical and socio-demographics collected	X	X
Randomisation	X	
Current medications	X	X
Comorbidities	X	X
ePRO Health related-Quality of Life survey. Instruments used as follows:<br>1. EORTC-QLQ-C30 (30 items) ±<br>2. *Considering patients cancer type:*<br>• Cervical cancer: EORTC QLQ – CX24 (24 items)<br>• Endometrial cancer: EORTC QLQ – EN24 (24 items)<br>• Breast cancer: EORTC QLQ - BR23 (23 items)<br>3. EQ-5D-5L (5 items) (measures QALY)<br>4. Appraisal Self-Care Agency-R (15 items) (measures power to self-care)	X	X
ePRO Patient-reported symptom questionnaire includes -PROMIS and PRO-CTCAE items<br>Fear of cancer recurrence, Adherence	X	X
Body weight	X	X
BIA measurement	x	x
Hand-grip strength	x	x
Diet intake assessments<br>(multiple 24hour dietary recalls & food frequency questionnaire)	x	x
Diet quality assessment<br>(World Cancer Research Fund / American Institute for Cancer research score)	x	x
Use of resources form		X
End of study patient questionnaire—includes system usability, satisfaction scales		X
Clinician/stakeholder questionnaires		X
Qualitative interviews with patients, healthcare professionals and wider team (process evaluation)		X
Clinician record of actions/follow-up at survivorship and symptom management encounters<br>Data extracted from hospital records		X
Drop out date, reason (data collected as required)		

a. Baseline measures may be completed up to 2 weeks after enrollment and prior to attendance at the initial clinic visit. Electronic survey reminders will be sent to patients to facilitate ePRO completion

Note: Clinical follow-up will be according to standard of care. Follow-up visits are not specifically required per protocol other than those needed for routine clinical care.

6.0 Statistical Plan

Once data entry is finalized, study data will be assessed for incompatible, discrepant or clinically implausible values. Outlying values for all distributions, in isolation and over time, will be identified. Any concerning data will be reconciled against original source data. Following completion of cleaning the database will be locked and the randomization code will be released.

The study sample will be described in detail. Continuous variables will be described by their means and SDs, medians and IQRs, and their range; while categorical variables will be described by their counts and percentages in each category.

Feasibility outcomes will be similarly described. These include the number of enrolled patients who complete the baseline and follow-up ePRO surveys, and the number of patients who partake in the healthcare professional consultation after ePRO data triggers such as a review/consultation; the number of patients that require medical review and the timeframe to Medical review; the average consultation time; the number of patients enrolled in the clinic; extra Health Care Professional time required and resources required for the intervention; and reasons for not completing the intervention will be collected through Drop Out Forms and with qualitative discussions with HCP involved in the clinic.

Between-group patient-relevant outcome differences, which can be used to inform a subsequent efficacy trial, will generally be estimated using generalized linear models with link functions based on the outcome for a given model, and a random effect for patient when relevant (e.g. longitudinally measured outcomes). All model-based estimates will be reported with 95% CIs and exact p-values. Missing data will be evaluated, and based on what we observe, dealt with in whatever manner we find appropriate based on current best practices. All analyses will be conducted using the R Project for Statistical Computing and the RStudio IDE. All trial reporting will be done following CONSORT and the CONSORT addendum for pilot/feasibility trials (Eldridge et al, 2016)

All analyses will be conducted and/or supervised by the HRB CRF-C Principal Statistician (Dr Darren Dahly), under their established quality systems and SOPs, and in accordance with *ICH E9 Statistical Principles for Clinical Trials* and *ICH E6 Good Clinical Practices.*

7 .0 Safety and Adverse Events

7.1 Recording of Adverse Events

It is not anticipated that any study related adverse events will occur during the study. This is an observational project involving collection of data regarding breast and gynaecological cancer care administered in accordance with this protocol. Information related to toxicities from adjuvant therapy will be collected as described above through ePRO measures, but these will not be considered adverse events. We do not anticipate any adverse events associated with completion of the ePRO modules. Patients will have the option of skipping questions they do not wish to answer. In event that study participation does result in any event that has negative consequences for the subject, this will be recorded by the PI in the EDC (Electronic Data Capture) and will be reported to the Sponsor and Ethics committee.

8.0 Data Handling and Record Keeping

8.1 Confidentiality

Study data will be maintained in a dedicated database in a secure location. A unique study ID number will be assigned to each subject. This will be linked to the patient identifying information by a list maintained by the PI in a secure location, separate to other study documentation. This is primarily to facilitate subsequent follow-up of patients. The patient identity list will remain at the clinical site and will not be forwarded to any third party, including the EDC platform. It will be available for review by the study monitor, auditors or inspectors as required. University College Cork, as study Sponsor, is the data controller for the research database.

8.2 Source Documentation

Electronic and/or paper case report form (CRF) will be used as source documentation in this project.

8.3 Case Report Forms

Data will be entered onto study specific eCRFs. With the EDC platform, almost all actions performed by users are tracked. It is possible to review what each user did in any field. Any missing data will be explained. If a data point cannot be answered due to missing data or other known reason, the EDC platform allows for this to be noted, any item on the CRF left blank because the procedure was not performed will be denoted by "N/D". Items not applicable to the individual case, will be recorded as "N/A". All entries should be printed legibly in black ink where paper used. In correcting any error, a single straight line will be drawn through the incorrect entry

without obscuring the original entry and the correct entry recorded above it. All such changes must be initialed and dated as per ICH-Good Clinical Practice (GCP).

8.4 Records Retention

Study documents and data will be kept for at least 10 years and wsill thereafter either be destroyed or fully anonymized and retained, as scientific interests dictate at that time.

9.0 Ethical Considerations

This study will be conducted in accordance with the Declaration of Helsinki, the applicable sections of ICH GCP , and the terms of approval of the responsible Ethics Committee, the Clinical Research Ethics Committee (CREC) of Cork Teaching Hospitals. Full ethical approval was granted by the CREC on March 1st, 2021. If any data breach is detected during the study, it will be reported in <72 hours to the Data Protection Commission (DPC).

10.0 Study Finances

10.1 Funding Source:

The costs of this study will be funded by an Irish Cancer Society grant (WHI19CON) and a grant from Breakthrough Cancer Research. Patients will not be paid for taking part in the study and will be attending on their regular clinic schedule other than the baseline clinic visit.

10.2 Indemnity for the performance of the study

Protocol and prototype indemnity will be provided by UCC. Malpractice indemnity will be provided to research staff as part of Clinical Indemnity Scheme coverage provided by the States Claims Agency.

11.0 Sponsorship

The study will be sponsored by UCC.

12.0 References

Allison, K. H., Hammond, M. E. H., Dowsett, M., McKernin, S. E., Carey, L. A., Fitzgibbons, P. L., et al. (2020). Estrogen and progesterone receptor testing in breast cancer: ASCO/CAP guideline update. PMID: 31928404; DOI: 10.1200/JCO.19.02309

Buchholz I, Janssen MF, Kohlmann T, Feng YS (2018). A systematic review of studies comparing the measurement properties of the three-level and five-level versions of the EQ-5D. Pharmacoeconomics 36(6):645-661.

Cella, D., Choi, S., Garcia, S., Cook, K. F., Rosenbloom, S., Lai, J. Set al. (2014). Setting standards for severity of common symptoms in oncology using the PROMIS item banks and expert judgment. Quality of Life Research: An International Journal of Quality of Life Aspects of Treatment, Care and Rehabilitation, 23, 2651–2661. http://dx.doi.org/ 10.1007/s11136-014-0732-6

Cessna, J. M., Jim, H. S., Sutton, S. K., Asvat, Y., Small, B. J., Salsman, J. M. & Perez, L. (2016). Evaluation of the psychometric properties of the PROMIS Cancer Fatigue Short Form with cancer patients. Journal of psychosomatic research, 81, 9-13.

Chamney PW, Wabel P, Moissl UM, Müller MJ, Bosy-Westphal A, Korth O, Fuller NJ (2007) A whole-body model to distinguish excess fluid from the hydration of major body tissues. *Am J Clin Nutr*. Jan;85(1):80-9.

Damschroder LJ, Aron DC, Keith RE, Kirsh SR, Alexander JA, Lowery JC. (2009) Fostering implementation of health services research findings into practice: a consolidated framework for advancing implementation science. Implement Sci.4(1):50.

EORTC core and disease specific and symptom specific tools and associated publications are available at https://qol.eortc.org/

Ferguson M, Capra S, Bauer J, Banks M. Development of a valid and reliable malnutrition screening tool for adult acute hospital patients. Nutrition. 1999 Jun;15(6):458-64

Fess EE (1986). The need for reliability and validity in hand assessment instruments. *J Hand Surg Am*, 11(5).

Government of Ireland (2017) National Cancer Strategy 2017-2026. Government of Ireland: Dublin. Available at: http://health.gov.ie/wp-content/uploads/2017/07/National-Cancer-Strategy-2017-2026.pdf

Hegarty J, Murphy A, Hanan T, O' Mahony M, Landers M, McCarthy B, Lehane E, Noonan B, Fitzgerald S, Reidy M, Saab M, Corrigan M, Mullen L. (2018). Acute Sector Cancer Survivorship Services in the Irish Context: a mixed method scoping study. National Cancer Control Programme: Dublin

Institute of Medicine (2005) From Cancer Patient to Cancer Survivor: Lost in Transition. Washington, DC, National Academies Press.

Kluetz PG, Chingos DT, Basch EM, Mitchell SA. Patient-reported outcomes in cancer clinical trials: measuring symptomatic adverse events with the National Cancer Institute's Patient-Reported Outcomes version of the Common Terminology Criteria for Adverse Events (PRO-CTCAE). Am Soc Clin Oncol Educ Book. 2016; 35:67-73.

Kouwenberg, C. A., Kranenburg, L. W., Visser, M. S., Busschbach, J. J., & Mureau, M. A. (2019). The validity of the EQ-5D-5L in measuring quality of life benefits of breast reconstruction. Journal of Plastic, Reconstructive & Aesthetic Surgery, 72(1), 52-61.

Lebel, S., Simard, S., Harris, C., Feldstain, A., Beattie, S., McCallum, M., ... & Devins, G. M. (2016). Empirical validation of the English version of the Fear of Cancer Recurrence Inventory. Quality of Life Research, 25(2), 311-321.

Lewis, J. R. (1995). IBM computer usability satisfaction questionnaires: Psychometric evaluation and instructions for use. International Journal of Human–Computer Interaction, 7(1), 57–78.

May, C., Finch, T., Mair, F., Ballini, L., Dowrick, C., Eccles, M., Gask, L., MacFarlane, A., Murray, E., Rapley, T., Rogers, A., Treweek, S., Wallace, P., Anderson, G., Burns, J. and Heaven, B. (2007) 'Understanding the implementation of complex interventions in health care: the normalization process model', BMC Health Services Research 7: 148.

Mierzynska J, Taye M, Pe M, Coens C, Martinelli F, Pogoda K, Velikova G, Bjelic-Radisic V., Cardoso F, Brain E, Ignatiadis M, Piccart M., Rutgers E, Van Tienhoven G., Mansel R, Wildiers H., Bottomley A. (2019) Reference Values for the EORTC QLQ-C30 in Early and Metastatic Breast Cancer. Eur J Cancer 2019. 125 (2020): 69-82.

Mullen, L. Hanan, T. National Cancer Survivorship Needs Assessment: Living with and beyond cancer in Ireland. National Cancer Control Programme: Dublin. ISBN 978-1-78602-133-5

Mullen, L. Hanan, T. National Cancer Survivorship Needs Assessment: Living with and beyond cancer in Ireland. National Cancer Control Programme: Dublin.

National Cancer Registry Ireland (NCRI) (2017) Cancer Factsheet Overview & most common cancers [Accessed 10th April 2018] available at https://www.ncri.ie/factsheets

National Cancer Strategy 2017-2026. (2017) Government of Ireland: Dublin.

National Coalition for Cancer Survivorship (2018). Examples of Cancer Care Plans [Accessed 25th April 2018] available at https://www.canceradvocacy.org/resources/planning-your-care/examples-of-cancer-care-plans/

National Comprehensive Cancer Network. (2018). NCCN distress thermometer and problem list for patients. Available at https://www.nccn.org/patients/resources/life_with_cancer/pdf/nccn_distress_thermometer.pdf .

O'Connor, M., Drummond, F., O'Donovan, B., & Donnelly, C. (2019) The Unmet Needs of Cancer Survivors in Ireland: A Scoping Review. National Cancer Control Programme; Dublin.

PROMIS® (Patient-Reported Outcomes Measurement Information System) Available at: Accessed at https://www.healthmeasures.net/explore-measurement-systems/promis#:~:text=PROMIS%C2%AE%20(Patient%2DReported%20Outcomes,individuals%20living%20with%20chronic%20conditions

Richards, M., Corner, J., Maher, J. (2014) The National Cancer Survivorship Initiative: new and emerging evidence on the ongoing needs of cancer survivors. British Journal of Cancer. 2011:105:S1-S4.

Shams-White MM, Brockton NT, Mitrou P, Romaguera D, Brown S, Bender A, Kahle LL, Reedy J (2019). Operationalizing the 2018 World Cancer Research Fund/American Institute for Cancer Research (WCRF/AICR) Cancer Prevention Recommendations: A Standardized Scoring System. Nutrients, Jul;12;11(7):1572.

Sharma, P., Dunn, R. L., Wei, J. T., Montie, J. E., & Gilbert, S. M. (2016). Evaluation of point-of-care PRO assessment in clinic settings: integration, parallel-forms reliability, and patient acceptability of electronic QOL measures during clinic visits. Quality of Life Research, 25(3), 575-583.

Sharp, J. L., Gough, K., Pascoe, M. C., Drosdowsky, A., Chang, V. T., & Schofield, P. (2018). The modified Memorial Symptom Assessment Scale Short Form: a modified response format and rational scoring rules. Quality of Life Research, 27(7), 1903-1910.

Sousa, V. D., Zauszniewski, J. A., Bergquist-Beringer, S., Musil, C. M., Neese, J. B., & Jaber, A. A. F. (2010). Reliability, validity and factor structure of the Appraisal of Self-Care Agency Scale–Revised (ASAS-R). Journal of evaluation in clinical practice, 16(6), 1031-1040.

Teare, M. D., Dimairo, M., Shephard, N., Hayman, A., Whitehead, A., & Walters, S. J. (2014). Sample size requirements to estimate key design parameters from external pilot randomised controlled trials: a simulation study. Trials, 15(1), 264. https://trialsjournal.biomedcentral.com/articles/10.1186/1745-6215-15-264

Van Leeuwen M., Husson O., Alberti P., Arraras J., Chinot O., Costantini A., Darlington A.S., Dirven L., Eichler M., Hammerlid E., Holzner B., Johnson C., Kontogianni M., Kjaer T., Morag O., Nolte S., Nordin A., Pace A., Pinto M., Polz K., Ramage J., Reijneveld J., Serpentini S., Tomaszewski K., Vassiliou V., Verdonck - de Leeuw I., Vistad I., Young T., Aaronson N., van de Poll-Franse L., on behalf of the EORTC Quality of Life Group. Understanding the quality of life (QOL) issues in survivors of cancer: towards the development of an EORTC QOL cancer survivorship questionnaire. Health Qual Life Outcomes 2018; 16 (1):114

Viechtbauer, W., Smits, L., Kotz, D., Budé, L., Spigt, M., Serroyen, J., & Crutzen, R. (2015). A simple formula for the calculation of sample size in pilot studies. Journal of clinical epidemiology, 68(11), 1375-1379. PMID: 26146089; DOI: 10.1016/j.jclinepi.2015.04.014

Whitehead, A. L., Julious, S. A., Cooper, C. L., & Campbell, M. J. (2016). Estimating the sample size for a pilot randomised trial to minimise the overall trial sample size for the external pilot and main trial for a continuous outcome variable. Statistical methods in medical research, 25(3), 1057-1073. https://doi.org/10.1177/0962280215588241

Whitehead, A. L., Sully, B. G., & Campbell, M. J. (2014). Pilot and feasibility studies: is there a difference from each other and from a randomised controlled trial? Contemporary clinical trials, 38(1), 130-133. DOI:https://doi.org/10.1016/j.cct.2014.04.001

Wolff, A. C., Hammond, M. E. H., Allison, K. H., Harvey, B. E., Mangu, P. B., Bartlett, J. M., et al (2018). Human epidermal growth factor receptor 2 testing in breast cancer: American Society of Clinical Oncology/College of American Pathologists clinical practice guideline focused update. Archives of pathology & laboratory medicine, 142(11), 1364-1382. PMID: 29846122; DOI: 10.1200/JCO.2018.77.8738

World Cancer Research Fund/American Institute for Cancer Research. Continuous update project expert report 2018. Recommendation and public health and policy implications. Available at dietandcancerreport.org

13.0 Study Documents Versions Log

IMPORTANT NOTE: The following documents have their own version and date and have been ethics approved as individual documents.

Study Title: Women's Cancer Survivorship: The LYSA (Linking You to support and Advice) Trial

Short Title: The LYSA Trial **Study Reference:** WHI19CON **Sponsor:** University College Cork (UCC) **UCC code:** 19137

**CREC REFERENCE:** ECM 4 (y) 20/10/2020	**First ethics approval** **Sent:** 26 Nov 2020 **Approval Date:** 01 Dec 2020 **CREC rev ref:** ECM 3 (ddd) 08/12/2020	**Amendment #1** **Sent:** 11 Jan 2021 **Approval Date:** 20 Jan 2021 **CREC rev ref:** ECM 3 (r) 09/02/2021	**Amendment #2** **Sent:** 16 Feb 2021 **Approval Date:** 01 Mar 2021 **CREC rev ref:** ECM 3 (fff) 09/03/2021	**Amendment #3** **Sent:** 16 Apr 2021 **Approval Date:** 5 May 2021 **CREC rev ref:** ECM 3 (ww) 11/05/2021	**Amendment #4** **Sent:** 21 Sep 2021 **Approval Date:** 12 October 2021 **CREC rev ref:** ECM 3 (qqq) 19/10/2021	**Amendment #5** **Sent:** 22 Feb 2022 **Approval Date:** 09 Mar 2022 **CREC rev ref:** ECM 3 (l) 05/04/2022	**Amendment #6** **Sent:** **Approval Date:** **CREC rev ref:**
Protocol	V3 of 26 Nov 2020	V4 of 08 Jan 2021	V5 of 16 Feb 2021	V6 of 16 Apr 2021	V7 of 21 Sep 2021	**V8 of 22 Feb 2022**	**V9 of 06 Jan 2023**
App 1a. Survey for Ongoing Monitoring of Symptoms and concerns	V1 of 04 Sep 2020	V2 of 08 Jan 2021	/	/	**V2 of 21 Sep 2021**		
App 1b. Triggers for Clinic Visit and their Calculation	V1 of 14 Sep 2020	**V2 of 08 Jan 2021**					
App 2a. Pre QOL Survey	V1 of 04 Sep 2020	V2 of 08 Jan 2021	/	**V3 of 16 Apr 2021**			
App 2b. Post QOL survey	V1 of 04 Sep 2020	V2 of 08 Jan 2021	/	**V3 of 16 Apr 2021**			
App 3a. Dietetics Food Frequency questionnaire	V1 of 13 Sep 2020	**V2 of 16 Dec 2020**					
App 3b. Dietetics Acceptance / Non-acceptance criteria for dietetic clinic	/	**V1 of 16 Dec 2020**					
App 3c. Dietitian Clinic Record Form	/	V1 of 08 Jan 2021	**V2 of 16 Feb 2021**				

App 4a. Feasibility and satisfaction survey	V1 of 03 Nov 2020	/	/	/	**V2 of 21 Sep 2021**		
App 4b. Interview schedule for feasibility process evaluation (for patients and HCPs)	V1 of 03 Nov 2020	/	/	/	**V2 of 21 Sep 2021**		
App 5. Nurse Record Form	V1 of 13 Sep 2020	**V2 of 8 Jan 2021** Minor change V2.1 of 19 Apr 2021 (format modification and rewording questions)					
App 6. Patient information leaflet and Consent form for participation in the trial Participant Information Leaflet-Patients Informed Consent Form	V2 of 26 Nov 2021	V3 of 8 Jan 2021	**V4 of 16 Feb 2021**				**V5 of 06 Jan 2023 (amended to include consent to contact GP and updated sponsor DPN)**
App 7. Participant Information Leaflet-HCP Informed Consent Form	V1 of 24 Nov 2021	V2 of 8 Jan 2021	**V3 of 16 Feb 2021**				
App 8. GP letter	/	/	V1 of 16 Feb 2021	**V2 of 16 Apr 2021**			
App 9a. Care Plan Breast Cancer	/	/	**V1 of 16 Feb 2021**				
App 9b. Care Plan Gynae Cancer	/	/	**V1 of 16 Feb 2021**				
App 10a. Instructions ePRO Castor	/	/	**V1 of 16 Feb 2021**				
App 10b. Instructions T-Pro patient information	/	/	**V1 of 16 Feb 2021**				
App 11. Patient recruitment advertisements	/	/	V1 of 16 Feb 2021	**V2 of 16 Apr 2021**			

App 12. Study patient diaries	/	/	**V1 of 16 Feb 2021**				
App 13a. Nurse-Dietitian Clinic Schedule&Instructions-Intervention	/	/	V1 of 16 Feb 2021<br>Minor change V1.1 of 13 May 2021 (inclusion bullet 5 page 2)	/	**V2 of 21 Sep 2021**		
App 13b. Nurse-Dietitian Clinic Schedule&Instructions-Control	/	/	V1 of 16 Feb 2021<br>Minor change V1.1 of 13 May 2021 (inclusion bullet 4 page 2)	/	**V2 of 21 Sep 2021**		
App 14a. Pathway Structure	/	/	**V1 of 16 Feb 2021**				
App 14b. Fatigue-Pathway	/	/	**V1 of 16 Feb 2021**				
App 14c. Hot Flashes Pathway	/	/	**V1 of 16 Feb 2021**				
App 14d. FOR Pathway	/	/	**V1 of 16 Feb 2021**				
App 14e. Sleep Pathway	/	/	**V1 of 16 Feb 2021**				
App 14f. Vaginal Discomfort Pathway	/	/	**V1 of 16 Feb 2021**				
App 14g. Cognition Pathway	/	/	**V1 of 16 Feb 2021**				
App 14h. Emotional Distress Pathway	/	/	**V1 of 16 Feb 2021**				
App 14i. Joint Pain Pathway	/	/	**V1 of 16 Feb 2021**				
App 14j. Pain Pathway	/	/	**V1 of 16 Feb 2021**				
App 14k. Bowel Pathway	/	/	**V1 of 16 Feb 2021**				
App 14. Urinary Pathway l	/	/	**V1 of 16 Feb 2021**				
App 14m. Sexual Health Pathway	/	/	**V1 of 16 Feb 2021**				

App 14n. Swelling Pathway	/	/	**V1 of 16 Feb 2021**				
