# Supplementary material for: A complex survivorship intervention utilizing electronic patient-reported outcomes in breast and gynecologic Cancer: the linking you to support and advice [LYSA] trial
Source: Breast. 2026 Feb 19;86:104740. doi: 10.1016/j.breast.2026.104740 (PMC12966741; doi:10.1016/j.breast.2026.104740)
Supplement: Supplementary Figure S1 [file mmc2.pptx]

## Slide 1
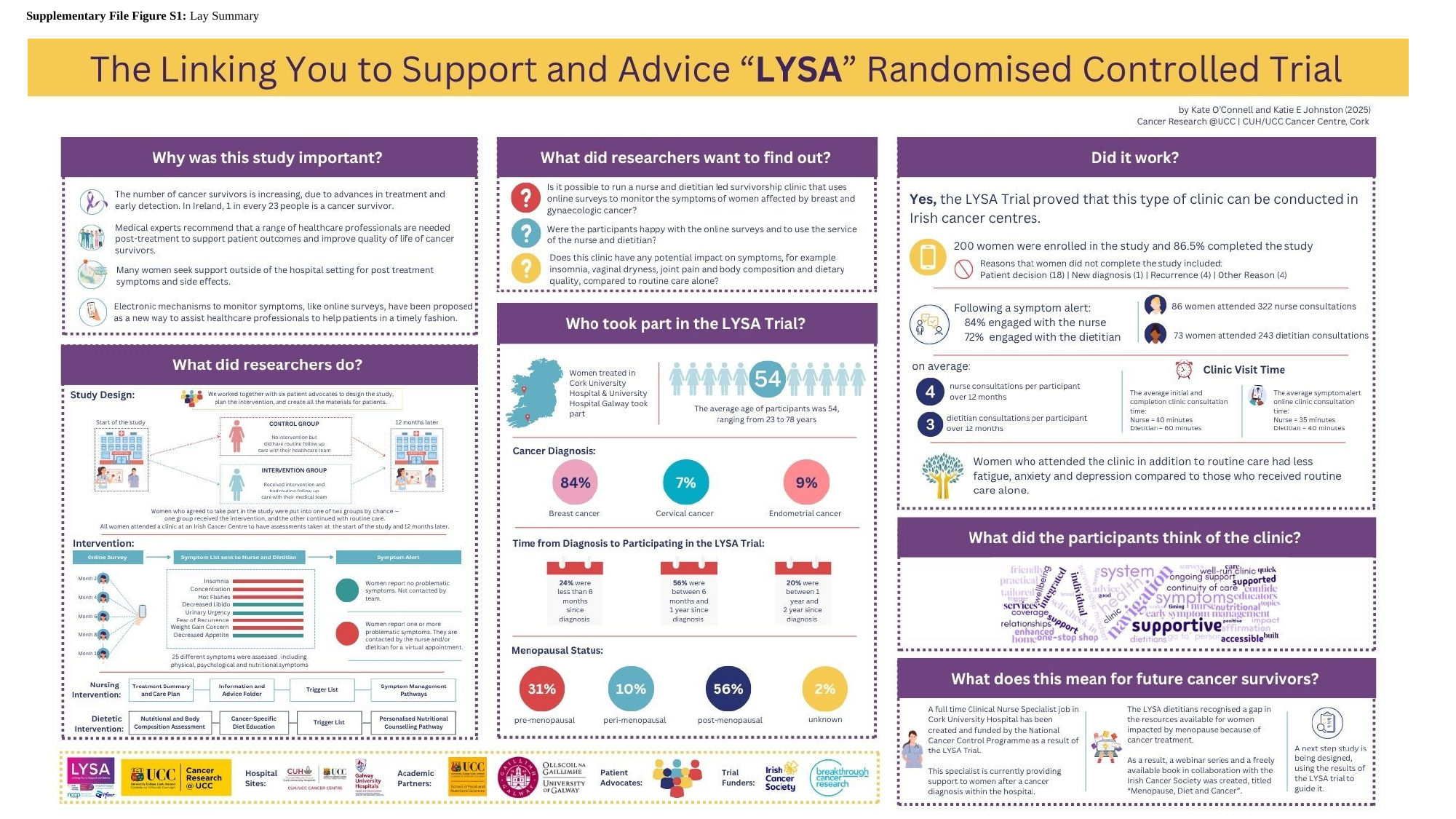

Supplementary File Figure S1: Lay Summary

## Slide 2
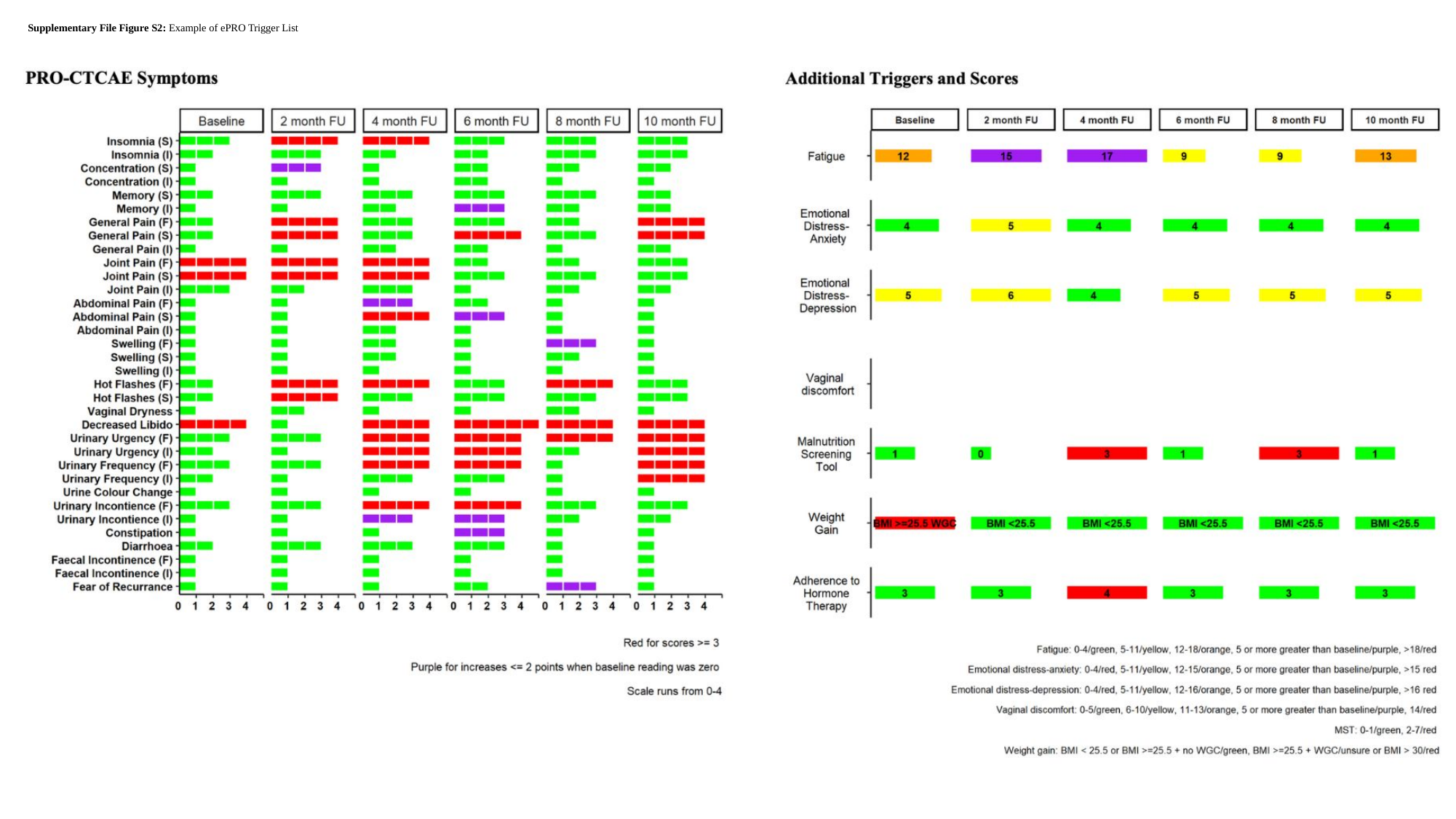

Supplementary File Figure S2: Example of ePRO Trigger List
