# Supplementary material for: A complex survivorship intervention utilizing electronic patient-reported outcomes in breast and gynecologic Cancer: the linking you to support and advice [LYSA] trial
Source: Breast. 2026 Feb 19;86:104740. doi: 10.1016/j.breast.2026.104740 (PMC12966741; doi:10.1016/j.breast.2026.104740)
Supplement: Supplementary Table S2 [file mmc4.docx]

**Supplementary Table S2:** Interview/Focus Group schedules* for: (a) Provider interviewees - Health Care Professionals/Members of the Wider Research Team/Stakeholders (b) Intervention Group Patients (c) Control Group Patients

| **Provider interviewees – Healthcare Professionals/Members of the Wider**  **Research Team/Stakeholders/ PPI representatives** | **Experimental Arm Patient Interviewees** | **Active Comparator Arm Patient Interviewees** |
| --- | --- | --- |
| Can you talk to me about your overall personal experience of participating in the LYSA trial? | Can you talk to me about your overall personal experience of participating in the LYSA trial? | Can you talk to me about your overall personal experience of participating in the LYSA trial? |
| Can you briefly describe your role in relation to the trial? | What were the main elements of the LYSA intervention which worked best for you? And  Why? (Probe: one-to-one sessions with the clinic’s nurse and dietitian, treatment summary and care plan, help/advice/education/counselling re: diet/nutrition, regular monitoring of your symptoms (bimonthly ePROs), help with managing your symptoms/adverse effects/concerns, personalized symptom management pathway plan(s))? | What were the main elements of the LYSA intervention which worked best for you? And  Why? (Probe: one-to-one sessions with the clinic’s nurse and dietitian, treatment summary and care plan) |
| In your opinion was the intervention delivered as originally intended and if not, please describe the main adaptations made to suit the local practice context? | What were the main elements of the LYSA intervention which did not work well for you?  And why? | What were the main elements of the LYSA intervention which did not work well for you?  And why? |
| Opinions on the main components of the intervention (probed for each component)? | In terms of the timing of this LYSA intervention, when do you think that  post-treatment services such as those provided through this study and clinic ideally should be offered to patients with cancer? | In terms of the timing of this LYSA intervention, when do you think that post-treatment services such as those provided through this study and clinic ideally should be offered to patients with cancer? |
| What were the main components of the intervention which did/did not work well in terms of facilitating implementation of LYSA/the survivorship clinic? | For approximately what length of time do you think that patients with cancer would benefit from post-treatment services such as those provided through this study and clinic? | For approximately what length of time do you think that patients with cancers would benefit from post-treatment services such as those provided through this study and clinic? |
| Do you have any proposals in terms of possible solutions to problems encountered with regards to implementing the intervention | What were your experiences of using the web-based system of surveys (ePROs) on a 2-monthly basis? | What were your experiences of using the web-based system of surveys (ePROs) at the beginning and end of the study? |
| What are your reflections on issues of nonparticipation and participant drop-outs from the study, in terms of reasons why and what could be done to improve study accrual and attrition rates in future  studies? | Was the additional follow-up care and support you have received from the healthcare professionals in the women’s survivorship clinic as part of participating in the study over past 12 months beneficial? (Expand/probe on benefits) | Was the support you received as part of the study, in addition to your usual routine follow-up care over past 12 months beneficial (please describe)? (Probe clinic visits at baseline and end of study) |
| From what you know about LYSA, is there anything you would like to change? | Is there anything you would change about the current LYSA intervention, based on your personal experience? | Is there anything you would change about the current LYSA intervention, based on your personal experience? |
| If you could influence the design and planning for future cancer survivorship clinics/services - what are your views as to what those clinics/services would entail? | If you could influence the design and planning for future cancer survivorship clinics /services what would they look like? | If you could influence the design and planning for future cancer survivorship clinics/services what would they look like? |
| For approximately what length of time do you think that patients with cancer would benefit from post-treatment services such as those provided through this study? | Would feedback in the form of a graph/chart etc. based on the information from the ePRO surveys you completed on a two-monthly basis have been something you would have been interested in  throughout the period of the trial? | Any other reflections on the implementation of LYSA trial? |
| Any other reflections on the implementation and sustainability of LYSA study and LYSA intervention? Probe factors influencing implementation and maintenance of LYSA? | Any other reflections on the implementation and sustainability of LYSA trial and LYSA intervention? |  |

*Probes and paraphrasing were used in conjunction with interview questions to elicit more detailed understanding of interviewee experiences.
