# Supplementary material for: A complex survivorship intervention utilizing electronic patient-reported outcomes in breast and gynecologic Cancer: the linking you to support and advice [LYSA] trial
Source: Breast. 2026 Feb 19;86:104740. doi: 10.1016/j.breast.2026.104740 (PMC12966741; doi:10.1016/j.breast.2026.104740)
Supplement: Supplementary Table S3 [file mmc5.docx]

**Supplementary Table S3:** Baseline Sociodemographic Characteristics

| **Characteristic** | **N** | **Overall** | **Active Comparator** | **Experimental** |
| --- | --- | --- | --- | --- |
|  |  | N = 200 | N = 98 | N = 102 |
| **Sociodemographic Information** |  |  |  |  |
| *What is your civil status?* | 193 |  |  |  |
| Cohabiting |  | 15 (7.8%) | 5/95 (5.3%) | 10/98 (10%) |
| Divorced |  | 9 (4.7%) | 6/95 (6.3%) | 3/98 (3.1%) |
| Married |  | 118 (61%) | 54/95 (57%) | 64/98 (65%) |
| Separated |  | 11 (5.7%) | 7/95 (7.4%) | 4/98 (4.1%) |
| Single |  | 29 (15%) | 17/95 (18%) | 12/98 (12%) |
| Widowed |  | 11 (5.7%) | 6/95 (6.3%) | 5/98 (5.1%) |
| *Which of the following best describes you?* * | 193 |  |  |  |
| White Irish |  | 177 (92%) | 86/95 (91%) | 91/98 (93%) |
| Any other white background |  | 14 (7.3%) | 8/95 (8.4%) | 6/98 (6.1%) |
| Any other Asian background |  | 1 (0.5%) | 0/95 (0%) | 1/98 (1.0%) |
| Chinese |  | 1 (0.5%) | 1/95 (1.1%) | 0/98 (0%) |
| *Which best describes your current living arrangements?* | 193 |  |  |  |
| Living alone |  | 33 (17%) | 21/95 (22%) | 12/98 (12%) |
| Living with family |  | 93 (48%) | 46/95 (48%) | 47/98 (48%) |
| Living with others |  | 3 (1.6%) | 1/95 (1.1%) | 2/98 (2.0%) |
| Living with partner |  | 60 (31%) | 24/95 (25%) | 36/98 (37%) |
| Other (please specify) |  | 4 (2.1%) | 3/95 (3.2%) | 1/98 (1.0%) |
| *Do you regularly take care of ill, disabled, elderly relatives/friends aged 15 years of age or more?* | *193* |  |  |  |
| No |  | 160 (83%) | 84/95 (88%) | 76/98 (78%) |
| Yes |  | 33 (17%) | 11/95 (12%) | 22/98 (22%) |
| *What is your level of education?* | 193 |  |  |  |
| None at all |  | 1 (0.5%) | 0/95 (0%) | 1/98 (1.0%) |
| Primary level |  | 5 (2.6%) | 2/95 (2.1%) | 3/98 (3.1%) |
| Secondary level |  | 62 (32%) | 32/95 (34%) | 30/98 (31%) |
| Third level (college/university) |  | 125 (65%) | 61/95 (64%) | 64/98 (65%) |
| *Before cancer diagnosis, which of these descriptions best describes your household?* | *193* |  |  |  |
| Coping on present income |  | 67 (35%) | 33/95 (35%) | 34/98 (35%) |
| Difficult on present income |  | 8 (4.1%) | 4/95 (4.2%) | 4/98 (4.1%) |
| Living comfortably on present income |  | 108 (56%) | 54/95 (57%) | 54/98 (55%) |
| Rather not say |  | 4 (2.1%) | 1/95 (1.1%) | 3/98 (3.1%) |
| Very difficult on present income |  | 6 (3.1%) | 3/95 (3.2%) | 3/98 (3.1%) |
| *At the moment, which of the following best defines your economic status?* | *193* |  |  |  |
| Employed |  | 57 (30%) | 26/95 (27%) | 31/98 (32%) |
| Looking after home/family |  | 19 (9.8%) | 8/95 (8.4%) | 11/98 (11%) |
| Other |  | 4 (2.1%) | 3/95 (3.2%) | 1/98 (1.0%) |
| Retired |  | 34 (18%) | 17/95 (18%) | 17/98 (17%) |
| Student |  | 3 (1.6%) | 1/95 (1.1%) | 2/98 (2.0%) |
| Unable to work due to COVID restrictions |  | 1 (0.5%) | 0/95 (0%) | 1/98 (1.0%) |
| Unable to work due to sickness or disability |  | 68 (35%) | 35/95 (37%) | 33/98 (34%) |
| Unemployed, looking for a job |  | 4 (2.1%) | 3/95 (3.2%) | 1/98 (1.0%) |
| Unemployed, not looking for a job |  | 3 (1.6%) | 2/95 (2.1%) | 1/98 (1.0%) |
| *Do you smoke regularly?* | 193 |  |  |  |
| No |  | 178 (92%) | 88/95 (93%) | 90/98 (92%) |
| Yes |  | 15 (7.8%) | 7/95 (7.4%) | 8/98 (8.2%) |
| *Do you drink alcohol?* | 193 |  |  |  |
| No |  | 44 (23%) | 21/95 (22%) | 23/98 (23%) |
| Yes |  | 149 (77%) | 74/95 (78%) | 75/98 (77%) |

* Standard reference classification used by the Central Statistics Office (Republic of Ireland)
