# Supplementary material for: A complex survivorship intervention utilizing electronic patient-reported outcomes in breast and gynecologic Cancer: the linking you to support and advice [LYSA] trial
Source: Breast. 2026 Feb 19;86:104740. doi: 10.1016/j.breast.2026.104740 (PMC12966741; doi:10.1016/j.breast.2026.104740)
Supplement: Supplementary Table S4 [file mmc6.docx]

**Supplementary File Table S4:** Thematised Reasons for Study Discontinuation

| **Theme** | **Reasons Given for Discontinuation** **(n=18)** |
| --- | --- |
| Lost to follow up / declined to come back to hospital | Failed to answer any contact made by research team to complete her end of study virtual dietetic assessment |
|  | Unhappy to travel to the hospital. Did Baseline survey and then did not want to return to hospital. Very nervous with covid initially and then very low mood * |
|  | Declined to attend the hospital for the end of study visit, completed end of study survey and then lost contact with the study |
|  | Patient did not disclose as lost contact with the study |
|  | Did not get to speak with patient in relation to drop out. Did not answer phone calls/texts or complete surveys |
|  | Patient declined to attend the hospital for the 12-month clinical visit due to work commitments *  Offered to reschedule etc. but patient did not respond |
|  | Declined to complete end of study survey and attend End of study hospital visit |
|  | Did not complete two consecutive surveys |
| Co-Morbidities | Unwell with co-morbidity (Ulcerative colitis) |
|  | Due to co-morbidities, she finds coming to the hospital difficult, wheelchair user |
|  | Ongoing comorbidity, multiple hospital appointments around this. Mood low at present- recently changed antidepressants by GP |
| IT issues | Daughter unable to help with IT |
|  | Son unable to help with IT |
| Wanting to move beyond cancer diagnosis | Did not want to be reminded of hospitals or cancer |
|  | Too busy back at work and unable to get time off for clinic visit. Feels well overall |
|  | Too busy to do the study |
|  | Personal decision |
| Change to treatment plan | Not meeting eligibility, not on/going on endocrine therapy |
| Dissatisfaction with randomisation | Part of control group, not happy with same |

** Some reasons articulated cross multiple themes reflecting the complex factors influencing participation in supportive care interventions.*

*Abbreviations: GP, General Practitioner; IT, Information Technology*
