# Supplementary material for: A complex survivorship intervention utilizing electronic patient-reported outcomes in breast and gynecologic Cancer: the linking you to support and advice [LYSA] trial
Source: Breast. 2026 Feb 19;86:104740. doi: 10.1016/j.breast.2026.104740 (PMC12966741; doi:10.1016/j.breast.2026.104740)
Supplement: Supplementary Table S5 [file mmc7.docx]

**Supplementary Table S5:** Completion Rates for ePRO Symptom Survey Package

| **Study Timepoint** | **Active Comparator (n = 98)** | | | | | | **Experimental (n = 102)** | | | | | |
| --- | --- | --- | --- | --- | --- | --- | --- | --- | --- | --- | --- | --- |
|  | Scheduled | Completed | | % | | | Scheduled | | Completed | | % | |
| **Baseline (T0)** | 98 | 95 | | 97 | | | 102 | | 98 | | 96 | |
| **Follow-Up 1 (T2)** |  |  | |  | | | 102 | | 95 | | 93 | |
| **Follow-Up 2 (T4)** |  |  | |  | | | 102 | | 94 | | 92 | |
| **Follow-Up 3 (T6)** |  |  | |  | | | 102 | | 92 | | 90 | |
| **Follow-Up 4 (T8)** |  |  | |  | | | 102 | | 91 | | 89 | |
| **Follow-Up 5 (T10)** |  |  | |  | | | 102 | | 85 | | 83 | |
| **End of the Study (T12)** | 98 | 87 | | 90 | | | 102 | | 91 | | 89 | |
|  |  | |  | |  |  | |  | |  | |  |
