# Supplementary material for: A complex survivorship intervention utilizing electronic patient-reported outcomes in breast and gynecologic Cancer: the linking you to support and advice [LYSA] trial
Source: Breast. 2026 Feb 19;86:104740. doi: 10.1016/j.breast.2026.104740 (PMC12966741; doi:10.1016/j.breast.2026.104740)
Supplement: Supplementary Table S6 [file mmc8.docx]

**Supplementary Table S6: LYSA ePRO Trigger Alert Matrix and Example**

| **Instruments** | **Raw Score** | **T score equivalence** | **Colour** | **Trigger** |
| --- | --- | --- | --- | --- |
| PRO-CTCAE™ Symptoms Term + Fear of recurrence | 0 to 2 or No | - |  | Trigger for **severity** alert: score ≥3 or increase in score by ≥2 compared to |
|  | 3 to 4 or Yes | - |  |  |

| PROMIS - Fatigue | 0-4 | Less than 39.7 |  | Trigger for **fatigue** alert: T score ≥ 70 and/or increase in T score of ≥ 5 compared to baseline score |
| --- | --- | --- | --- | --- |
|  | 5 to 11 | More than 39.7 to less than 57.0 |  |  |
|  | 12 to 18 | Equal to 57.0 and less than 69 |  |  |
|  | More than 18 | Equal or more than 69 |  |  |

| PROMIS - Emotional distress- anxiety | 0-4 | Less than 48 |  | Trigger for Emotional distress – **anxiety** alert: T score ≥ 70 and/or increase in T score of ≥ 5 compared to baseline score |
| --- | --- | --- | --- | --- |
|  | 5 to 11 | More than 48 to less 63.4 |  |  |
|  | 12 to 15 | Equal to 63.4 and less than 70 |  |  |
|  | More than 15 | Equal or more than 70 |  |  |

| PROMIS - Emotional distress- depression | 0-4 | Less than 49.0 |  | Trigger for Emotional distress- **depression** alert: T score ≥ 70 and/or increase in T score of ≥ 5 compared to baseline score |
| --- | --- | --- | --- | --- |
|  | 5 to 11 | More than 49.0 to less 62.2 |  |  |
|  | 12 to 16 | Equal to 62.2 and less than 70 |  |  |
|  | More than 16 | Equal or more than 70 |  |  |

| PROMIS – Vaginal Discomfort | 0 to 5 | Less than 48.09 |  | Trigger for **vaginal discomfort** alert: T score ≥ 70 and/or increase in T score of ≥ 5 compared to baseline score |
| --- | --- | --- | --- | --- |
|  | 6 to 10 | Equal or more than 48.09 to less than 58.56 |  |  |
|  | 11 to 13 | Equal to 58.56 and less than 69.81 |  |  |
|  | 14 | Equal or more than 69.81 |  |  |

| Malnutrition Screening Tool Score | 0 to 1 | - |  | Trigger if ≥2 |
| --- | --- | --- | --- | --- |
|  | 2 to 7 | - |  |  |

| Body Mass Index and Weight Gain Concern | - | 18.5 to less than 25.5 |  | Trigger if:  - BMI scores between 25.5 (included) to 30 (included) and they answer “yes” to weight gain concern  - BMI more than 30 |
| --- | --- | --- | --- | --- |
|  | - | More than 25.5 to 30 + no weight gain concern |  |  |
|  | - | More than 25.5 to 30 + weight gain concern |  |  |
|  | - | More than 30 |  |  |

| Adherence to hormonal therapy | 1 to 3 | - |  | Trigger if ≥4 |
| --- | --- | --- | --- | --- |
|  | 4 to 6 | - |  |  |

| Medication changes | No | - |  | Trigger if “Yes” |
| --- | --- | --- | --- | --- |
|  | Yes | - |  |  |

| Use of resources | No | - |  | Trigger if “Yes” |
| --- | --- | --- | --- | --- |
|  | Yes | - |  |  |

| Medical conditions | No | - |  | Trigger if “Yes” |
| --- | --- | --- | --- | --- |
|  | Yes | - |  |  |

| Hospitalisations | No | - |  | Trigger if “Yes” |
| --- | --- | --- | --- | --- |
|  | Yes | - |  |  |

*Abbreviations: BMI, Body Mass Index; PRO-CTCAE, Patient-Reported Outcomes version of the Common Terminology Criteria for Adverse Events; PROMIS, Patient-Reported Outcomes Measurement Information System*
