# Supplementary material for: A complex survivorship intervention utilizing electronic patient-reported outcomes in breast and gynecologic Cancer: the linking you to support and advice [LYSA] trial
Source: Breast. 2026 Feb 19;86:104740. doi: 10.1016/j.breast.2026.104740 (PMC12966741; doi:10.1016/j.breast.2026.104740)
Supplement: Supplementary Table S7 [file mmc9.docx]

**Supplementary Table S7:** Disease Specific EORTC QLQ Scores

| **Standardized EORTC-BR23 breast cancer scores at baseline and the end of the study (n=147)** | **N** | **Baseline (T0)** | |  | **Endpoint (T12)** | |
| --- | --- | --- | --- | --- | --- | --- |
|  |  | **Control**  N = 73*1* | **Active**  N = 74*1* | **N** | **Control**  N = 73*1* | **Active**  N = 74*1* |
| EORTC QLQ-BR23 body image score | 142 | 61 (31);  67 [33, 83] | 56 (33);  67 [25, 83] | 139 | 70 (29);  75 [50, 100] | 65 (28);  67 [50, 83] |
| *Missing values* |  | *2* | *3* |  | *6* | *2* |
| EORTC QLQ-BR23 sexual functioning score | 142 | 17 (18);  17 [0, 33] | 14 (18);  0 [0, 17] | 139 | 17 (20);  17 [0, 33] | 19 (19);  17 [0, 33] |
| *Missing values* |  | *2* | *3* |  | *6* | *2* |
| EORTC QLQ-BR23 sexual enjoyment score | 55 | 47 (28);  33 [33, 67] | 54 (31);  67 [33, 67] | 59 | 56 (25);  67 [33, 67] | 46 (28);  33 [33, 67] |
| *Missing values* |  | *41* | *51* |  | *48* | *40* |
| EORTC QLQ-BR23 future perspective score | 142 | 45 (26);  67 [33, 67] | 41 (31);  33 [0, 67] | 139 | 45 (29);  33 [33, 67] | 54 (30);  67 [33, 67] |
| *Missing values* |  | *2* | *3* |  | *6* | *2* |
| EORTC QLQ-BR23 systemic therapy side effects score | 142 | 75 (20);  81 [71, 86] | 74 (19);  76 [62, 90] | 139 | 77 (21);  86 [71, 90] | 82 (14);  86 [76, 90] |
| *Missing values* |  | *2* | *3* |  | *6* | *2* |
| EORTC QLQ-BR23 breast symptoms score | 142 | 74 (20);  75 [67, 83] | 71 (20);  75 [58, 83] | 139 | 81 (18); 83 [75, 100] | 83 (15); 83 [75, 92] |
| *Missing values* |  | *2* | *3* |  | *6* | *2* |
| EORTC QLQ-BR23 arm symptoms score | 142 | 78 (20);  78 [67, 100] | 80 (21);  89 [67, 100] | 139 | 80 (24);  89 [67, 100] | 82 (15);  78 [78, 100] |
| *Missing values* |  | *2* | *3* |  | *6* | *2* |
| EORTC QLQ-BR23 upset by hair loss score | 39 | 33 (34);  33 [0, 67] | 45 (41);  67 [0, 67] | 21 | 42 (41);  50 [0, 67] | 70 (35);  67 [67, 100] |
| *Missing values* |  | *57* | *51* |  | *61* | *65* |
| *1* Mean (SD); Median [Q1, Q3] |  |  |  |  |  |  |

| **Standardized EORTC QLQ-CX24 cervical cancer scores at baseline and the end of the study (n=11)** | **N** | **Baseline (T0)** | | **End of Study (T12)** | | | |
| --- | --- | --- | --- | --- | --- | --- | --- |
|  |  | **Control**  N = 4*1* | **Active**  N = 7*1* | **Control**  N = 4*1* | | **Active**  N = 7*1* | |
| EORTC QLQ-CX24 symptom experience score | 11 | 81 (8);  80 [76, 86] | 84 (9);  82 [82, 91] | 87 (8);  86 [82, 92] | | 87 (10);  88 [82, 94] | |
| EORTC QLQ-CX24 body image score | 11 | 72 (21);  67 [56, 89] | 67 (29);  67 [33, 89] | 72 (33);  78 [44, 100] | | 73 (32);  89 [56, 100] | |
| EORTC QLQ-CX24 sexual functioning score | 2 | 83 (NA);  83 [83, 83] | 58 (NA);  58 [58, 58] | 91.7 (NA);  91.7 [91.7, 91.7] | | 83.3 (NA);  83.3 [83.3, 83.3] | |
| *Missing values* |  | *3* | *6* | *3* | | *6* | |
| EORTC QLQ-CX24 lymphoedema score | 11 | 83 (19);  83 [67, 100] | 86 (18);  100 [67, 100] | 100 (0);  100 [100, 100] | | 81 (26);  100 [67, 100] | |
| EORTC QLQ-CX24 peripheral neuropathy score | 11 | 83 (19);  83 [67, 100] | 86 (26);  100 [67, 100] | 100 (0);  100 [100, 100] | | 76 (16);  67 [67, 100] | |
| EORTC QLQ-CX24 menopausal symptom score | 11 | 67 (27);  67 [50, 83] | 38 (49);  0 [0, 100] | 75 (17);  67 [67, 83] | | 52 (42);  67 [0, 100] | |
| EORTC QLQ-CX24 sexual worry score | 11 | 75 (32);  83 [50, 100] | 57 (46);  67 [0, 100] | 75 (32);  83 [50, 100] | | 76 (37);  100 [67, 100] | |
| EORTC QLQ-CX24 sexual activity score | 11 | 8 (17);  0 [0, 17] | 10 (25);  0 [0, 0] | 8 (17);  0 [0, 17] | | 14 (38);  0 [0, 0] | |
| EORTC QLQ-CX24 sexual enjoyment score (baseline) | 2 | 33.3 (NA);  33.3 [33.3, 33.3] | 33.3 (NA);  33.3 [33.3, 33.3] | 66.7 (NA);  66.7 [66.7, 66.7] | | 66.7 (NA);  66. 7 [66.7, 66.7] | |
| *Missing values* |  | *3* | *6* | *3* | | *6* | |
| *1* Mean (SD); Median [Q1, Q3] | | | | |  | |  |

| **Standardized EORTC QLQ-EN24 endometrial cancer scores at baseline and the end of the study (n=13)** | **N** | **Baseline (T0)** | | **End of Study (T12)** | | | |
| --- | --- | --- | --- | --- | --- | --- | --- |
|  |  | **Control**  N = 5*1* | **Active**  N = 8*1* | **Control**  N = 5*1* | | | **Active**  N = 8*1* |
| EORTC QLQ-EN24 lymphoedema score | 13 | 80 (30);  100 [67, 100] | 85 (30);  100 [83, 100] | 70 (25);  67 [67, 83] | | | 90 (13);  100 [83, 100] |
| *Missing values* |  | *0* | *0* | *0* | | | *1* |
| EORTC QLQ-EN24 urological symptom score | 13 | 65 (12);  67 [58, 67] | 77 (19);  79 [67, 92] | 78 (13);  83 [75, 83] | | | 79 (16);  83 [67, 92] |
| *Missing values* |  | *0* | *0* | *0* | | | *1* |
| EORTC QLQ-EN24 gastrointestinal symptom score | 13 | 83 (24);  93 [87, 93] | 79 (13);  77 [70, 93] | 91 (6);  87 [87, 93] | | | 89 (13);  93 [87, 93] |
| *Missing values* |  | *0* | *0* | *0* | | | *1* |
| EORTC QLQ-EN24 body image score | 13 | 70 (41);  83 [67, 100] | 69 (19);  67 [67, 75] | 90 (15);  100 [83, 100] | | | 83 (22);  100 [67, 100] |
| *Missing values* |  | *0* | *0* | *0* | | | *1* |
| EORTC QLQ-EN24 sexual problems score (baseline) | 5 | 72 (8);  72 [67, 78] | 74 (13);  67 [67, 89] | 94 (8);  94 [89, 100] | | | 93 (13);  100 [78, 100] |
| *Missing values* |  | *3* | *5* | *3* | | | *5* |
| EORTC QLQ-EN24 back pain score | 13 | 93 (15);  100 [100, 100] | 88 (17);  100 [67, 100] | 87 (18);  100 [67, 100] | | | 86 (26);  100 [67, 100] |
| *Missing values* |  | *0* | *0* | *0* | | | *1* |
| EORTC QLQ-EN24 tingling score | 13 | 87 (18);  100 [67, 100] | 63 (45);  83 [17, 100] | 53 (38);  67 [33, 67] | | | 67 (38);  67 [33, 100] |
| *Missing values* |  | *0* | *0* | *0* | | | *1* |
| EORTC QLQ-EN24 joint pain score | 13 | 87 (18);  100 [67, 100] | 71 (21);  67 [67, 83] | 67 (41);  67 [67, 100] | | | 71 (30);  67 [33, 100] |
| *Missing values* |  | *0* | *0* | *0* | | | *1* |
| EORTC QLQ-EN24 hair loss score | 13 | 40 (55);  0 [0, 100] | 63 (52);  100 [0, 100] | 73 (43);  100 [67, 100] | | | 100 (0);  100 [100, 100] |
| *Missing values* |  | *0* | *0* | *0* | | | *1* |
| EORTC QLQ-EN24 taste change symptom score | 13 | 60 (43);  67 [33, 100] | 88 (17);  100 [67, 100] | 73 (28);  67 [67, 100] | | | 100 (0);  100 [100, 100] |
| *Missing values* |  | *0* | *0* | *0* | | | *1* |
| EORTC QLQ-EN24 sexual interest score | 13 | 20 (30);  0 [0, 33] | 13 (17);  0 [0, 33] | 27 (37);  0 [0, 67] | | | 19 (26);  0 [0, 33] |
| *Missing values* |  | *0* | *0* | *0* | | | *1* |
| EORTC QLQ-EN24 sexual activity score | 13 | 27 (43);  0 [0, 33] | 13 (17);  0 [0, 33] | 27 (37);  0 [0, 67] | | | 19 (26);  0 [0, 33] |
| *Missing values* |  | *0* | *0* | *0* | | | *1* |
| EORTC QLQ-EN24 sexual enjoyment score | 5 | 50 (24);  50 [33, 67] | 22 (19);  33 [0, 33] | 83 (24);  83 [67, 100] | | | 67 (33);  67 [33, 100] |
| *Missing values* |  | *3* | *5* | *3* | | | *5* |
| *1* Mean (SD); Median [Q1, Q3] | | | | |  |  | |
