# Supplementary material for: A complex survivorship intervention utilizing electronic patient-reported outcomes in breast and gynecologic Cancer: the linking you to support and advice [LYSA] trial
Source: Breast. 2026 Feb 19;86:104740. doi: 10.1016/j.breast.2026.104740 (PMC12966741; doi:10.1016/j.breast.2026.104740)
Supplement: Supplementary Table S8 [file mmc10.docx]

**Supplementary File Table S8: Usability and Satisfaction Survey**

| **Level of agreement with statements in relation to participant experience of using the web-based system for completing electronic surveys (electronic Patient Reported Outcomes - ePROs). *** | **N** | **Overall** | | **Active Comparator** | | **Experimental Arm** | |
| --- | --- | --- | --- | --- | --- | --- | --- |
|  |  | N = 149 (%) | | N = 69 (%) | | N = 80 | |
| **The computer interface (screen layout) was pleasant and user friendly** | 149 |  | |  | |  | |
| Disagree |  | 4 | *(2.7%)* | 2 | *(2.9%)* | 2 | *(2.5%)* |
| Neither disagree nor agree |  | 5 | *(3.4%)* | 3 | *(4.3%)* | 2 | *(2.5%)* |
| Agree |  | 140 | *(94.0%)* | 64 | *(92.8%)* | 76 | *(95.0%)* |
| **The computer system was simple to use** | 149 |  |  |  |  |  | |
| Disagree |  | 7 | *(4.7%)* | 3 | *(4.3%)* | 4 | *(5.0%)* |
| Neither disagree nor agree |  | 5 | *(3.4%)* | 5 | *(7.2%)* | 0 | *(0.0%)* |
| Agree |  | 137 | *(91.9%)* | 61 | *(88.4%)* | 76 | *(95.0%)* |
| **I felt comfortable using the computerized questionnaire system** | 149 |  |  |  |  |  | |
| Disagree |  | 8 | *(5.4%)* | 4 | *(5.8%)* | 4 | *(5.0%)* |
| Neither disagree nor agree |  | 5 | *(3.4%)* | 5 | *(7.2%)* | 0 | *(0.0%)* |
| Agree |  | 136 | *(91.3%)* | 60 | *(87.0%)* | 76 | *(95.0%)* |
| **Overall, I was satisfied with completing questionnaires on the computerized system** | 149 |  |  |  |  |  | |
| Disagree |  | 6 | *(4.0%)* | 3 | *(4.3%)* | 3 | *(3.8%)* |
| Neither disagree nor agree |  | 5 | *(3.4%)* | 5 | *(7.2%)* | 0 | *(0.0%)* |
| Agree |  | 138 | *(92.6%)* | 61 | *(88.4%)* | 77 | *(96.3%)* |
| **I would prefer to complete computerized versions of questionnaires over paper-based versions** | 149 |  |  |  |  |  | |
| Disagree |  | 45 | *(30.2%)* | 23 | *(33.3%)* | 22 | *(27.5%)* |
| Neither disagree nor agree |  | 20 | *(13.4%)* | 10 | *(14.5%)* | 10 | *(12.5%)* |
| Agree |  | 84 | *(56.4%)* | 36 | *(52.2%)* | 48 | *(60.0%)* |
| **The information (such as on-line help, on-screen messages and other documentation) provided within this system was clear** | 149 |  |  |  |  |  | |
| Disagree |  | 3 | *(2.0%)* | 1 | *(1.4%)* | 2 | *(2.5%)* |
| Neither disagree nor agree |  | 8 | *(5.4%)* | 8 | *(11.6%)* | 0 | *(0.0%)* |
| Agree |  | 138 | *(92.6%)* | 60 | *(87.0%)* | 78 | *(97.5%)* |
| **In general, the time it took me to complete the computerized questionnaires on the ePRO system was reasonable** | 149 |  |  |  |  |  | |
| Disagree |  | 3 | *(2.0%)* | 1 | *(1.4%)* | 2 | *(2.5%)* |
| Neither disagree nor agree |  | 7 | *(4.7%)* | 5 | *(7.2%)* | 2 | *(2.5%)* |
| Agree |  | 139 | *(93.3%)* | 63 | *(91.3%)* | 76 | *(95.0%)* |
| ** Responses from a five-point Likert scale were collapsed into three categories: 'agree' (combining strongly agree and somewhat agree), 'neither disagree nor agree,' and 'disagree' (combining somewhat disagree and strongly disagree).* | | | | | | | |

| **Level of satisfaction with core elements of LYSA intervention in the Experimental Arm, over the past 12 months:** | **N** | **N = 79 (%)** | |  |
| --- | --- | --- | --- | --- |
| **The assessments of my symptoms, quality of life, and diet/nutrition** | 79 |  |  |  |
| Disagree |  | 0 | *(0.0%)* |  |
| Neither disagree nor agree |  | 1 | *(1.3%)* |  |
| Agree |  | 78 | *(98.7%)* |  |
| **The Treatment Summary and Care Plan I received at the first Clinic visit** | 79 |  |  |  |
| Disagree |  | 0 | *(0.0%)* |  |
| Neither disagree nor agree |  | 1 | *(1.3%)* |  |
| Agree |  | 78 | *(98.7%)* |  |
| **The information and advice I received to manage my symptoms/concerns** | 79 |  |  |  |
| Disagree |  | 0 | *(0.0%)* |  |
| Neither disagree nor agree |  | 0 | *(0.0%)* |  |
| Agree |  | 79 | *(100.0%)* |  |
| **The access to the care/services I needed to deal with my symptoms/concerns** | 79 |  |  |  |
| Disagree |  | 0 | *(0.0%)* |  |
| Neither disagree nor agree |  | 6 | *(7.6%)* |  |
| Agree |  | 73 | *(92.4%)* |  |
| **The timeliness of access to follow-up care/support services I received** | 79 |  |  |  |
| Disagree |  | 0 | *(0.0%)* |  |
| Neither disagree nor agree |  | 8 | *(10.1%)* |  |
| Agree |  | 71 | *(89.9%)* |  |
| **The level of follow-up support I received from the Clinic’s healthcare providers** | 79 |  |  |  |
| Disagree |  | 0 | *(0.0%)* |  |
| Neither disagree nor agree |  | 2 | *(2.5%)* |  |
| Agree |  | 77 | *(97.5%)* |  |
| **The one-to-one consultation sessions I had with the Clinic’s Nurse** | 79 |  |  |  |
| Disagree |  | 0 | *(0.0%)* |  |
| Neither disagree nor agree |  | 0 | *(0.0%)* |  |
| Agree |  | 79 | *(100.0%)* |  |
| **The one-to-one consultation sessions I had with the Clinic’s Dietitian** | 79 |  |  |  |
| Disagree |  | 1 | *(1.3%)* |  |
| Neither disagree nor agree |  | 0 | *(0.0%)* |  |
| Agree |  | 78 | *(98.7%)* |  |
| **Individualised nutritional advice/counselling I received from the Clinic’s Dietitian** | 79 |  |  |  |
| Disagree |  | 0 | *(0.0%)* |  |
| Neither disagree nor disagree |  | 3 | *(3.8%)* |  |
| Agree |  | 76 | *(96.2%)* |  |
| **Using the computerised system of electronic surveys (ePROs) to collect symptom and quality of life information** | 79 |  |  |  |
| Disagree |  | 0 | *(0.0%)* |  |
| Neither disagree nor agree |  | 2 | *(2.5%)* |  |
| Agree |  | 77 | *(97.5%)* |  |
| **My overall experience of participating in this Women’s Survivorship Study** | 79 |  |  |  |
| Disagree |  | 0 | *(0.0%)* |  |
| Neither disagree nor agree |  | 0 | *(0.0%)* |  |
| Agree |  | 79 | *(100.0%)* |  |
| **My Personalized Symptom Pathway Plan(s)** | 79 |  |  |  |
| Disagree |  | 0 | *(0.0%)* |  |
| Neither disagree nor agree |  | 2 | *(2.5%)* |  |
| Agree |  | 77 | *(97.5%)* |  |
| ** Responses from a five-point Likert scale were collapsed into three categories: 'agree' (combining strongly agree and somewhat agree), 'neither disagree nor agree,' and 'disagree' (combining somewhat disagree and strongly disagree).* | | | |  |

| **Level of satisfaction with core elements of the LYSA Trial in the Active Comparator Arm, over the past 12 months:** | **N** | **N = 70***^1^* (%) | |
| --- | --- | --- | --- |
| **The assessments of my symptoms, quality of life, and diet/nutrition** | 70 |  |  |
| Disagree |  | 3 | *(4.3%)* |
| Neither disagree nor agree |  | 6 | *(8.6%)* |
| Agree |  | 61 | *(87.1%)* |
| **The Treatment Summary and Care Plan I received at the first Clinic visit** | 70 |  |  |
| Disagree |  | 2 | *(2.9%)* |
| Neither disagree nor agree |  | 8 | *(11.4%)* |
| Agree |  | 60 | *(85.7%)* |
| **The information and advice I received to manage my symptoms/concerns** | 70 |  |  |
| Disagree |  | 3 | *(4.3%)* |
| Neither disagree nor agree |  | 7 | *(10.0%)* |
| Agree |  | 60 | *(85.7%)* |
| **The access to the care/services I needed to deal with my symptoms/concerns** | 70 |  |  |
| Disagree |  | 6 | *(8.6%)* |
| Neither disagree nor agree |  | 9 | *(12.9%)* |
| Agree |  | 55 | *(78.6%)* |
| **The timeliness of access to follow-up care/support services I received** | 70 |  |  |
| Disagree |  | 5 | *(7.1%)* |
| Neither disagree nor agree |  | 13 | *(18.6%)* |
| Agree |  | 52 | *(74.3%)* |
| **The level of follow-up support I received as part of my usual follow up care** | 70 |  |  |
| Disagree |  | 3 | *(4.3%)* |
| Neither disagree nor agree |  | 14 | *(20.0%)* |
| Agree |  | 53 | *(75.7%)* |
| **Using the computerised system of electronic surveys (ePROs) to collect symptom and quality of life information** | 70 |  |  |
| Disagree |  | 0 | *(0.0%)* |
| Neither disagree nor agree |  | 9 | *(12.9%)* |
| Agree |  | 61 | *(87.1%)* |
| **My overall experience of participating in this Women’s Survivorship Study** | 70 |  |  |
| Disagree |  | 2 | *(2.9%)* |
| Neither disagree nor agree |  | 7 | *(10.0%)* |
| Agree |  | 61 | *(87.1%)* |
| ** Responses from a five-point Likert scale were collapsed into three categories: 'agree' (combining strongly agree and somewhat agree), 'neither disagree nor agree,' and 'disagree' (combining somewhat disagree and strongly disagree).* | | |  |
