# Supplementary material for: A complex survivorship intervention utilizing electronic patient-reported outcomes in breast and gynecologic Cancer: the linking you to support and advice [LYSA] trial
Source: Breast. 2026 Feb 19;86:104740. doi: 10.1016/j.breast.2026.104740 (PMC12966741; doi:10.1016/j.breast.2026.104740)
Supplement: Supplementary Table S9 [file mmc11.docx]

**Supplementary Table S9** Positive and negative implementation factors linked to implementation of the LYSA Feasibility Trial organised according to the PRISM (RE-AIM) framework

| **Dimension**  * | **Definition / Examples in current study** | **Category**  **(as per process evaluation data)** | **Positives/Enablers of LYSA Intervention** | **Negatives/Barriers to LYSA Intervention** |
| --- | --- | --- | --- | --- |
| **Perspectives on the intervention** | E.g. history with similar programs and associated services | Intervention | Able to learn from international experience of similar intervention and ePRO monitoring system (shared learning) | Symptom management pathways not fully developed, implemented or resourced for rare symptoms (e.g. peripheral neuropathy, weight management in oncology) |
| **Values and characteristics of Implementing Settings.**  **Multilevel partner, organizational characteristics**  **Implementer characteristics.**  **Patient characteristics** | Refers to characteristics that affect organizations’ ability to successfully adopt, implement intervention.  Refers to characteristics of implementers, their settings, intended patients | Partners and relationships | Strong professional relationships, MDT and partnership between hospital, community organisations, clinical research infrastructure and collaborators at the university.  Strong professional relationships between healthcare professionals and patients  Access to community-based support centres and structures enabled through signposting  Professional respect and enthusiasm for LYSA | Reliant on personal relationships and implementer’s knowledge of infrastructure and health care professionals active in cancer survivorship supportive care |
| **External environment- e.g. potential drivers of inequalities** | Refers to context of relevant policies, strategies, incentives, regulatory environment and community resources | Enabling national policy and context | Increased national focus on enabling post treatment support for patients with cancer and linking with community-based services | Formal links between community and acute oncology survivorship services not fully developed.  LYSA limited to patients with breast and gynaecological cancers  Limited digital and English language capabilities impeded participation |
|  |  | External influencing factors |  | COVID-19  Cyberattack of Irish health service during study |
| **Implementation and sustainability infrastructure** | Refers to characteristics such as adopter training and support, dedicated team for implementation, resources and plans for sustainability. | Implementation, resources and plans for sustainability | Dedicated LYSA team supported through once off funding  LYSA deemed feasible  Parallel economic evaluation | Feasibility study- need a full trial  Lack of funding and dedicated staff to maintain LYSA  LYSA is feasible, problem is meeting the demand |
|  |  | Infrastructure | Dedicated staff/human resource  Infrastructure- access to clinical space, Castor digital system | Dedicated staff and buy in from organisation and partner services not secured for continued roll out  Ability to link university-based research system and clinical systems is complex requires data sharing agreements and arrangements |
| **Reach** | The number, proportion, and representativeness of individuals who were willing to participate in the LYSA study and intervention | Patient related factors | Motivation to participate | The process of symptom i.e. regular reporting via ePRO, acted as a reminder of cancer adding to psychological distress |
|  |  | Recruitment to study | Identification of barriers to enrolment early on enhanced recruitment.  Engagement of all relevant stakeholders.  Public Patient Involvement  Virtual/tele connectivity | Initial narrow clinical eligibility criteria.  COVID-19 context required adaptations to accrual and consent processes  Limited number of patients with gynaecological cancer recruited  Limited enrolment of ethnic minorities |
|  |  | Digital capabilities | Digital capability of participants or supportive person | Limited digital capabilities  Didn’t have access to paper surveys for those with limited digital access or capability |
| **Effectiveness or efficacy** | Positive and negative factors impacting the implementation of the LYSA study and intervention | MDT intervention | Clinical assistance or clinical backup for the nurse led service | Not a 24/7 service  Limited psycho-oncology services e.g. access to counselling |
|  |  | Having symptom pathways | Having identified symptom management pathways, referral systems and identified clinic provides a structure to manage symptoms proactively | Lack of fully resourced pathways for rarer symptoms  Dietetic triggers need to be more developed as not meeting the needs or identifying the needs of BMI 18.5kg/m^2^ - 25.5 kg/m^2^ or BMI <18.5 kg/m^2^ |
|  |  | Ethics and reporting processes | Streamlined ethics application and reporting templates and processes for clinical studies across sites | Complexity of ethics applications in multisite studies |
|  |  | Service level support | Support with logistics  Support from clinical and department leaders especially line managers of staff involved  Support from hospital leadership team | Difficulties integrating digital intervention into clinical workflow |
|  |  | Patient level affirmation of the benefits of the intervention through feedback, ePROs and PPI | Positive experiences with clinic (e.g. supportive, accessible, helped with symptoms, good set up, accommodating) |  |
| **Adoption** | Enablers and barriers to the initial adoption of the intervention among participants + those involved in delivering the intervention | Co-design | Involvement of PPI.  Support of the academic and wider research team was an enabler.  Steering group of stakeholders.  Being part of a broader multicentre “Women’s Health Initiative” |  |
|  |  | Being a research study | Research allows learning to be integrated into practice.  Ability to get publications is also positive |  |
| **Implementation (individual and setting)** | Enablers and barriers in implementing the intervention as planned over the two years of the study from recruitment to completion of study | Having a clear study protocol | Team developed protocol  Following the protocol very well  Being able to make minor changes as needed to the protocol- as the real-world scenario becomes clearer | Implementing a complex intervention in real world settings leads to unanticipated barriers and challenges |
| **Maintenance (individual and setting)** | Activities to be executed to sustain the (implementation of the) intervention over time beyond the current study | Funding and resources | Access to funding and resources for staff to support enhanced supportive care clinics and systems  Time and resources to manage workload associated with reviewing and responding to survey initiated triggers | Sustainment of a clinical intervention beyond a research study when research funding is limited, and posts are contract posts  Embedding research into clinical roles with clinicians having conflicting responsibilities |
|  |  | Digital infrastructure | Digitally enabled infrastructure- linking to existing systems in the hospital and into the community | Digital infrastructure developed during research is not easily embedded into routine care of the patient outside of the research study  Difficulty embedding LYSA digital record into patient records |
|  |  | Giving live feedback to patient and HCP | Graphs and triggers live to patient and HCP is recommended. | Feedback is not automated- requires HCP response and takes time. |

** Deductive analysis was applied in this analysis providing a structured method of analysis that applied predefined themes or dimensions drawn from the PRISM(RE_AIM) framework to the presentation of the qualitative data presented in this table.*

*Abbreviations: BMI, Body Mass Index; ePROs, electronic Patient Reported Outcomes; HCP, Healthcare Professional; MDT, Multidiscplinary Team; PPI, Patient and Public Involvement*
